# A subset of microRNAs in the *Dlk1‐Dio3* cluster regulates age‐associated muscle atrophy by targeting *Atrogin‐1*

**DOI:** 10.1002/jcsm.12578

**Published:** 2020-06-03

**Authors:** Yeo Jin Shin, Eun‐Soo Kwon, Seung‐Min Lee, Seon‐Kyu Kim, Kyung‐Won Min, Jae‐Young Lim, Bora Lee, Jae Sook Kang, Ju Yeon Kwak, Young Hoon Son, Jeong Yi Choi, Yong Ryul Yang, Seokho Kim, Yeon‐Soo Kim, Hak C. Jang, Yousin Suh, Je‐Hyun Yoon, Kwang‐Pyo Lee, Ki‐Sun Kwon

**Affiliations:** ^1^ Aging Research Center Korea Research Institute of Bioscience and Biotechnology (KRIBB) Daejeon Korea; ^2^ Personalized Genomic Medicine Research Center Korea Research Institute of Bioscience and Biotechnology (KRIBB) Daejeon Korea; ^3^ Department of Biology, College of Natural Sciences Gangneung‐Wonju National University Gangneung Korea; ^4^ Department of Rehabilitation Medicine Seoul National University Bundang Hospital Gyeonggi‐do Korea; ^5^ Department of Biomolecular Science, KRIBB School of Bioscience Korea University of Science and Technology (UST) Daejeon Korea; ^6^ Department of Functional Genomics, KRIBB School of Bioscience Korea University of Science and Technology (UST) Daejeon Korea; ^7^ Department of Medicinal Biotechnology, College of Health Sciences Dong‐A University Busan Korea; ^8^ Department of New Drug Discovery and Development Chungnam National University Daejeon Korea; ^9^ Internal Medicine Seoul National University Bundang Hospital Gyeonggi‐do Korea; ^10^ Department of Genetics Albert Einstein College of Medicine NY USA; ^11^ Department of Biochemistry and Molecular Biology Medical University of South Carolina Charleston SC USA

**Keywords:** *Dlk1‐Dio3* miRNA cluster, Muscle aging, Sarcopenia, Atrophy, Cachexia, Atrogin‐1, MiR‐376c‐3p

## Abstract

**Background:**

The microRNAs (miRNAs) down‐regulated in aged mouse skeletal muscle were mainly clustered within the delta‐like homologue 1 and the type III iodothyronine deiodinase (*Dlk1‐Dio3*) genomic region. Although clustered miRNAs are coexpressed and regulate multiple targets in a specific signalling pathway, the function of miRNAs in the *Dlk1‐Dio3* cluster in muscle aging is largely unknown. We aimed to ascertain whether these miRNAs play a common role to regulate age‐related muscle atrophy.

**Methods:**

To examine anti‐atrophic effect of miRNAs, we individually transfected 42 miRNA mimics in fully differentiated myotubes and analysed their diameters. The luciferase reporter assay using target 3′ untranslated region (UTR) and RNA pull‐down assay were employed to ascertain the target predicted by the TargetScan algorithm. To investigate the therapeutic potential of the miRNAs *in vivo*, we generated adeno‐associated virus (AAV) serotype 9 expressing green fluorescent protein (GFP) (AAV9‐GFP) bearing miR‐376c‐3p and infected it into the tibialis anterior muscle of old mice. We performed morphometric analysis and measured *ex vivo* isometric force using a force transducer. Human gluteus maximus muscle tissues (ages ranging from 25 to 80 years) were used to investigate expression levels of the conserved miRNAs in the *Dlk1‐Dio3* cluster.

**Results:**

We found that the majority of miRNAs (33 out of 42 tested) in the cluster induced anti‐atrophic phenotypes in fully differentiated myotubes with increasing their diameters. Eighteen of these miRNAs, eight of which are conserved in humans, harboured predicted binding sites in the 3′ UTR of muscle atrophy gene‐1 (*Atrogin‐1*) encoding a muscle‐specific E3 ligase. Direct interactions were identified between these miRNAs and the 3′ UTR of *Atrogin‐1*, leading to repression of Atrogin‐1 and thereby induction of eIF3f protein content, in both human and mouse skeletal muscle cells. Intramuscular delivery of AAV9 expressing miR‐376c‐3p, one of the most effective miRNAs in myotube thickening, dramatically ameliorated skeletal muscle atrophy and improved muscle function, including isometric force, twitch force, and fatigue resistance in old mice. Consistent with our findings in mice, the expression of miRNAs in the cluster was significantly down‐regulated in human muscle from individuals > 50 years old.

**Conclusions:**

Our study suggests that genetic intervention using a muscle‐directed miRNA delivery system has therapeutic efficacy in preventing Atrogin‐1‐mediated muscle atrophy in sarcopenia.

## Introduction

Aging skeletal muscle exhibits progressive loss of muscle mass, strength, and function, a condition collectively known as sarcopenia.^1^ Muscle mass decreases by approximately 1% every year starting at age 30,^2^, ^3^ and the prevalence of sarcopenia is approximately 10% in elderly individuals at age 60, and it increases to 50% at age 80.^4^, ^5^, ^6^ Because age‐related muscle wasting triggers not only disorders in physical activity but also several diseases, such as type 2 diabetes mellitus, obesity, dyslipidaemia, and hypertension, in the elderly,^7^ there is an urgent need to develop effective therapeutic agents for healthy muscle aging. However, to date, no drug for controlling sarcopenia has been approved by the Food and Drug Administration. In view of the recently established *International Classification of Diseases, Tenth Revision, Clinical Modification* disease code for sarcopenia^8^ by the World Health Organization, the development of diagnostic strategies and therapeutic agents against sarcopenia is expected to accelerate.

MicroRNAs (miRNAs), the most extensively studied class of noncoding RNAs, are single‐stranded molecules approximately 22 nucleotides (nt) in length that act as gene regulators at a posttranscriptional level.^9^ MiRNAs are frequently arranged in polycistronic clusters and coexpressed at a specific genomic locus, showing a general tendency to cotarget the same complex or pathway.^10^, ^11^, ^12^, ^13^, ^14^ We recently demonstrated that >50% of down‐regulated miRNAs in aged mouse skeletal muscle and myoblasts are clustered in the delta‐like homologue 1 and the type III iodothyronine deiodinase (*Dlk1‐Dio3*) imprinted genomic region.^15^, ^16^, ^17^ *Dlk1‐Dio3* is the largest known placental mammalian‐specific miRNA cluster. The majority of miRNAs are contained within the antisense *Rtl1* and the larger transcript of *Mirg*.^18^, ^19^, ^20^ Several miRNAs located in the *Dlk1‐Dio3* locus, including miR‐127,miR‐410,miR‐431,miR‐433, and miR‐434, are related to muscle homeostasis functions, such as myogenic differentiation and muscle regeneration.^16^, ^21^, ^22^, ^23^, ^24^ However, the specific function of the *Dlk1‐Dio3* miRNA cluster in muscle aging is largely unclear. In this study, we aimed to ascertain whether these collective miRNAs play a common role in age‐related muscle atrophy and thereby have therapeutic potential to treat sarcopenia.

Muscle mass is determined by a dynamic balance between anabolism and catabolism.^25^ Various stimuli, including interleukin‐1 (IL‐1), tumour necrosis factor‐alpha (TNF‐α), and glucocorticoids, activate catabolic pathways that induce muscle atrophy via up‐regulation of muscle‐specific E3 ligases, such as MuRF1 and Atrogin‐1.^26^ These E3 ligases are reported to be markedly increased not only in specific muscles upon disuse or nerve injury but also in several diseases, including diabetes, sepsis, hyperthyroidism, and cancer cachexia.^27^, ^28^, ^29^ Relative to other muscular disorders, the regulatory mechanism of E3 ligase expression in aging muscle remains largely unknown. Transcriptional levels of *MuRF1* and *Atrogin‐1* have been determined in aged muscle of mouse, rat, and human,^30^, ^31^, ^32^, ^33^, ^34^, ^35^, ^36^, ^37^, ^38^, ^39^, ^40^, ^41^, ^42^, ^43^ but the results obtained to date are controversial. Here, we have focused on elucidating the mechanism underlying the regulation of Atrogin‐1 expression by miRNAs during the muscle aging process. Our studies demonstrate that genetic intervention using miRNAs within the *Dlk1‐Dio3* cluster has a beneficial preventive effect on Atrogin‐1‐mediated atrophy in muscle aging.

## Material and methods

### Human skeletal muscle samples

Human skeletal muscle tissues (gluteus maximus muscle) obtained from patients who underwent total hip replacement arthroplasty at Seoul National University Bundang Hospital (SNUBH) were immediately placed in liquid nitrogen and stored at −70°C. The Institutional Review Board of SNUBH (B‐1710‐050‐009) approved this study. Written informed consent was obtained from participants or their legal guardians. In total, 20 muscle samples provided from patients of various ages (25, 27, 32, 33, 33, 41, 46, 46, 50, 50, 51, 55, 66, 67, 70, 71, 75, 79, 79, and 80 years) were used to assess the expression of miRNA. RNA was isolated from ~30 μg of human samples and was further purified using TRIzol (Invitrogen) for analysis of miRNA expression.

### Animal models

Young (3‐month‐old) and old (24‐month‐old) C57BL/6 mice were purchased from the Laboratory Animal Resource Center [Korea Research Institute of Bioscience and Biotechnology (KRIBB)]. All mice in this study were kept on a standard laboratory diet (3.1 kcal/g) purchased from Damul Science (Daejeon, Korea). To overexpress a mimic of miRNA in muscle tissues, 50 μL (1 × 10^10^ GC) of green fluorescent protein (GFP) expressing adeno‐associated virus (AAV)9‐Ctrl or miR‐376c‐3p (Applied Biological Materials Inc., Canada) was directly injected into the tibialis anterior (TA) muscle or contralateral muscle of each old mouse using a 29G (0.33‐mm) needle connected to an insulin syringe. Four weeks after injection, the AAV‐injected mice were sacrificed, and isolated muscle tissues were used for further analysis. To generate a cachexia mouse model, colon‐26 (C26) cells [5 × 10^5^ cells in 50 μL phosphate‐buffered saline (PBS)] were subcutaneously injected into BABL/c mice using an insulin syringe as described previously.^44^ One week before tumour cell inoculation, 50 μL (1 × 10^10^ GC) of either AAV9‐Control (AAV9‐Ctrl) or AAV9‐miR‐376‐3p was intramuscularly injected into TA or contralateral muscle. Mice were sacrificed on Day 14 after tumour inoculation for experimentation. Colon 26 cells (CLS Cell Lines Service) were cultured in RPMI1640 (Gibco) with amphotericin B–penicillin–streptomycin and 10% foetal bovine serum (FBS). Experiments with mice and viruses were performed according to established protocols approved by the Animal Care and Use Committee of KRIBB.

### Cell culture

Primary myoblasts were isolated from hind limb muscle as described previously.^45^ Briefly, muscle tissue was minced using scissors and incubated with dissociation buffer including Dispase II (2.4 U/mL, Roche), collagenase D (1%, Roche), and 2.5 μM of CaCl_2_ at 37°C for 20 min. The slurry was triturated using a serological pipette and subsequently passed through a 70‐μm nylon mesh (BD Biosciences) to remove debris. Cells were collected and resuspended in growth medium consisting of Ham's F‐10 (Gibco) supplemented with 20% FBS containing amphotericin B–penicillin–streptomycin and 5 ng/mL of basic fibroblastic growth factor. To eliminate fibroblasts, cells were plated on noncoated plates for 1 h, and floating cells were transferred to collagen‐coated culture dishes. Differentiation of primary myoblasts was induced by culturing cells in differentiation medium comprising Dulbecco's modified Eagle medium (DMEM) (Gibco) supplemented with antibiotics and 5% horse serum. C2C12 cells (American Type Culture Collection) were cultured in DMEM (Gibco) with amphotericin B–penicillin–streptomycin and 10% FBS. Differentiation was initiated 24 to 48 h after seeding by changing to differentiation medium [DMEM (Gibco) with amphotericin B–penicillin–streptomycin and 2% horse serum]. For dexamethasone‐induced atrophy, C2C12 cells were initially differentiated for 4 days, after which 100 μM of dexamethasone (Sigma‐Aldrich) was added into the medium, as described previously.^46^ Human skeletal muscle myoblasts (HSMMs; isolated from 17‐ or 19‐year‐old donors; Lonza Co.) were cultured in growth medium consisting of skeletal muscle basal medium 2 (Lonza) supplemented with gentamicin–amphotericin B, human epidermal growth factor, dexamethasone, l‐glutamine, and 10% FBS. Differentiation was initiated 24 to 48 h after seeding by incubating in DMEM/F12 (Gibco) with gentamicin–amphotericin B and 2% horse serum. For colon 26 conditioned media, colon 26 was cultured in DMEM (Gibco) with 10% FBS. After 72 h, the supernatant was collected and filtered through a 0.22 μm filter. C26 culture medium treatment was 50% in differentiation medium (DMEM with 2% horse serum).

### Transfection and luciferase assay

Mimics and inhibitors of miRNAs were purchased from *mir*Vana (Invitrogen) or *AccuTarget*™ (Bioneer) (*Tables* S2 and S3). Small interfering RNAs (siRNAs) are shown in *Table* S4. Mimics and inhibitors of miRNA and siRNA (50–100 nM each) were transfected into primary myoblasts, C2C12, or HSMMs using RNAiMAX (Invitrogen) according to the manufacturer's recommended protocols.

For luciferase assays, the full‐length 5598 nt 3′ untranslated region (UTR) of mouse *Atrogin‐1* mRNA was cloned into pmirGLO (Promega), in which the *luc2* coding sequence exists in the multicloning site, and the *hRluc‐neo* coding sequence was used as an internal control. The *Atrogin‐1* 3′ UTR mutant with deletion of the miR‐376c‐3p binding region (positions 3781–3787) was also cloned into the pmirGLO vector for the luciferase assay. 293T cells were transfected with 50 nM of miRNA mimic and luciferase plasmids (200 ng) using Lipofectamine 2000 (Invitrogen). At 48 h after transfection, cell lysates were used for the luciferase assay with the Dual‐Luciferase Reporter Assay System (Promega) and Victor X3 (Perkin Elmer).

### Quantitative reverse transcription–polymerase chain reaction and microRNA expression analysis

RNA preparation and cDNA synthesis were performed according to standard protocols. Quantitative reverse transcription–PCR (qRT‐PCR) was performed using StepOnePlus™ (Applied Biosystems) in a total reaction volume of 20 μL containing cDNA, primers, and SYBR Master Mix (Applied Biosystems). The primer sequences are listed in *Table* S5. The data were normalized to *Actb* or *GAPDH* mRNA levels in each reaction. For analysis of mature miRNA expression, TaqMan MiRNA Assays were performed according to the manufacturer's protocol (Applied Biosystems). qRT‐PCR was conducted in 96‐well plates with TaqMan Universal PCR Master Mix II (no uracil *N*‐glycosylase) and TaqMan Small RNA Assay Mix. The sequences of the small RNA‐specific forward PCR primer, specific reverse PCR primer, and small RNA‐specific TaqMan MGB probe are presented in *Table* S6. *U6* snRNA served as the endogenous control for normalization.

### Antisense oligonucleotide pull‐down analysis

For analysis of miRNA–mRNA interactions, a hybridization‐based strategy was utilized to purify target mRNAs associated with miRNAs. C2C12 cells were transfected with the indicated *firefly* luciferase reporter containing the wild‐type or deletion mutant miR‐376c‐3p binding site in the *Atrogin‐1* 3′ UTR. Cell lysates (1 mg) were incubated with 2 μg of biotinylated or nonbiotinylated antisense oligonucleotides (ASOs) (*Table* S7) designed to hybridize specifically to either the endogenous *Atrogin‐1* 3′ UTR or *Luciferase2* mRNA, which were incubated at 4°C for 3 h with rotation. Streptavidin‐agarose beads (Novagene) were added to the binding mixture and subsequently incubated at 4°C for 2 h. After the beads were washed three times with 1 mL of NT2 buffer (50 mM of Tris–HCl, pH 7.5, 150 mM of NaCl, 1 mM of MgCl_2_, and 0.05% NP‐40), complexes were incubated with 20 units of RNase‐free DNase I (15 min at 37°C) and further with 0.1% sodium dodecyl sulfate (SDS)/0.5 mg/mL proteinase K (15 min at 55°C) to remove DNA and proteins, respectively. RNA was isolated from the ASO pull‐down material via acidic phenol extraction and cDNAs synthesized from miRNA using the qScript miRNA cDNA Synthesis Kit (Quanta Biosciences) or total RNA with a random hexamer using Maxima Reverse Transcriptase according to the manufacturer's protocol. Next, cDNAs were assessed via qPCR analysis with SYBR (Kapa Biosystems) using the Bio‐Rad iCycler. Normalization of ASO pull‐down results was conducted by quantifying the relative levels of *U6* snRNA or *Gapdh* mRNA in each sample in parallel.

### Immunoblot analysis

Muscle tissues and isolated myoblasts were homogenized in lysis buffer (50 mM of Tris‐Cl, pH 7.4, 150 mM of NaCl, 0.5% Triton X‐100, 1 mM of EDTA, and 1 mM of MgCl_2_) containing protease and phosphatase inhibitors. Lysates were centrifuged at 15 000 × *g* for 20 min at 4°C, and the resulting supernatants were subjected to SDS–polyacrylamide gel followed by immunoblot analysis. Antibodies used for immunoblotting included those specific for ACTB (β‐actin; Abcam), ACTN1 (Santa Cruz Biotechnology), α‐tubulin (Santa Cruz Biotechnology), AKT (Santa Cruz Biotechnology), mTOR (Cell Signaling Technology), S6K (Cell Signaling Technology), 4EBP (Cell Signaling Technology), FOXO3a (Cell Signaling Technology), SMAD2/3 (Cell Signaling Technology), p‐AKT (Cell Signaling Technology), p‐S6K (Cell Signaling Technology), p‐4EBP (Cell Signaling Technology), p‐FOXO3a (Cell Signaling Technology), p‐SMAD2/3 (Cell Signaling Technology), MuRF1 (Santa Cruz Biotechnology), Atrogin‐1 (Thermo Scientific, ECM), and eIF3f (Novus). GAPDH was developed in our laboratory. ACTB, ACTN1, and α‐tubulin served as the endogenous controls for normalization.

### *Ex vivo* isometric force and fatigue measurements

Intact TA muscle‐tendon complexes were isolated from young and old mice and mounted vertically to a bath chamber containing carbogen‐(95% O_2_/5% CO_2_)‐saturated Krebs‐Ringer buffer (118 mM of NaCl, 4.75 mM of KCl, 24.8 mM of NaHCO_3_, 1.18 mM of KH_2_PO_4_, 2.5 mM of CaCl_2_ 2H_2_O, 1.18 mM of MgSO_4_, and 10 mM of glucose) at pH 7.4 and 25°C. The muscle was fixed to the bottom of the organ bath by a clamp, while the tendon was connected to a force transducer (AD Instruments, USA) by a string. The optimal muscle length (*L*_0_) was determined as the length producing the highest twitch force at supramaximal voltage (100 V for 1 ms) using a modified previous protocol.^47^, ^48^ Twitch forces (mN) were determined using an electrical stimulator (AD Instruments, USA) at 100 V and 1 Hz. Tetanus forces (mN) were determined at 100 V from 10 to 200 Hz. The isometric forces were normalized by the weight of TA muscle. For analysis of resistance to muscle fatigue, the muscle was repeatedly stimulated every 30 s for 10 min. Then, the isometric forces were analysed as a percentage of the initial maximal contractile force. All experiments were performed at room temperature (25°C) and analysed using LabChart software (AD Instruments, USA).

### Morphometric analysis

For immunostaining, differentiated C2C12 myotubes were fixed in 4% paraformaldehyde and incubated with 0.3% Triton X‐100 to enhance permeability. Fixed samples were blocked with 3% bovine serum albumin in PBS and treated with anti‐MyHC (Santa Cruz Biotechnology), followed by washing in PBS and incubation with AlexaFluor 488 (Invitrogen) secondary antibodies. For eosin staining, differentiated C2C12 myotubes were fixed in ice‐cold methanol at −20°C for 15 min and stained with Eosin Y (Thermo Scientific) for 15 min. Samples were washed three times with distilled water, and images were captured using a Nikon Eclipse Ti‐U microscope. For quantification of myotube diameters, four views were randomly selected. Diameters of myotubes on the selected views were calculated with microscope imaging software (NIS‐Elements Basic Research, Nikon). The protein:genomic DNA ratios were measured as described previously.^49^ Genomic DNA was isolated using a specific Genomic DNA Preparation Kit (NANOHELIX), and the protein concentrations of cell lysates were determined with the BCA Protein Assay Reagent (Pierce).

For immunohistochemical analysis, skeletal muscle tissues were fixed in 4% paraformaldehyde, and frozen section samples or paraffin‐embedded sections were prepared. The frozen sections (10 μm thick) were stained with 4′,6‐diamidino‐2‐phenylindole (DAPI) and antibody according to standard protocols. Antibodies for immunohistochemical analysis recognized laminin (Sigma‐Aldrich) and GFP (Invitrogen). The paraffin sections were stained with haemotoxylin and eosin according to standard protocols. For measurement of the cross‐sectional area, six views were randomly selected. The cross‐sectional areas on the views were calculated using NIH ImageJ software (http://rsb.info.nih.gov/ij).

### Statistical analysis

Quantitative data are presented as the mean ± SD, unless otherwise indicated. Differences between means were evaluated using Student's unpaired *t* test. *P* values < 0.05 were considered statistically significant.

## Results

### miRNAs in the *Dlk1‐Dio3* cluster increase myotube diameter

**FIGURE 1:**
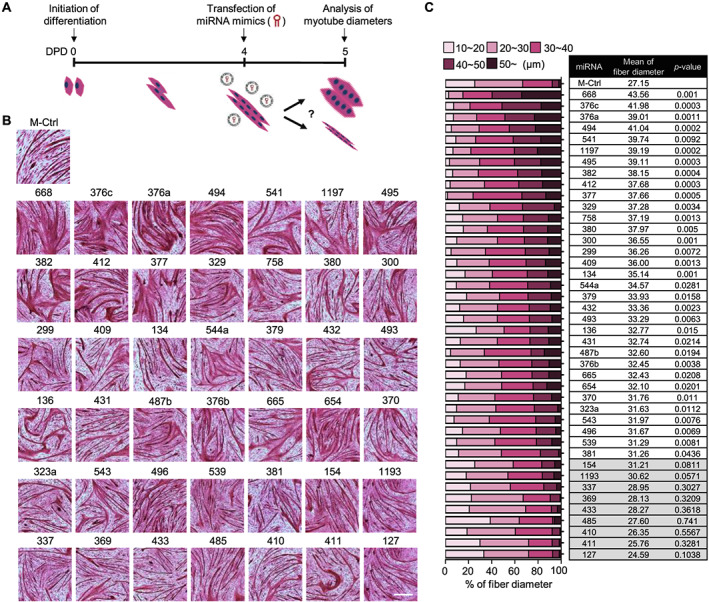
Age‐associated miRNAs in the *Dlk1‐Dio3* cluster increase the diameters of fully differentiated myotubes. (A) Scheme of screening of microRNAs (miRNAs) causing the muscle hypertrophic phenotype. At Day 4 after differentiation induction of C2C12 cells, miRNA mimics in the *Dlk1‐Dio3* cluster were individually transfected into fully differentiated myotubes. Myotube diameters were measured at 24 h after transfection. DPD, days post differentiation. (B) Representative images of differentiated myotubes transfected with the indicated miRNA mimics. The myotubes were stained with eosin Y for measurement of diameters. Scale bar, 50 μm. (C) Percentage of myotubes with different diameters after transfection with the indicated miRNA mimics. Darker colour represents a larger diameter. Four different fields were randomly selected for diameter measurements using microscope imaging software (NIS‐Elements Basic Research, Nikon). MiRNAs with no significant changes are shaded grey. The data are presented as the mean ± SD.

On the basis of our previous reports showing the down‐regulation of the *Dlk1‐Dio3* cluster of miRNAs in muscle aging,^15^, ^16^, ^17^ we investigated the possibility that the miRNAs in the cluster could regulate muscle hypertrophy or atrophy. Therefore, we examined whether 42 pre‐miRNAs, conserved in both mouse and human sequences, in the *Dlk1‐Dio3* locus are involved in the regulation of atrophy, a major phenotype of aged muscle. To this end, a mimic (M) of each pre‐miRNA was transfected into fully differentiated C2C12 myotubes, and its impact on myotube diameter was evaluated (*Figure* 1A). Remarkably, 33 of the 42 pre‐miRNAs examined induced significantly larger diameters than did the control miRNAs (*Figure* 1B and C), showing anti‐atrophic effects. These results suggested that age‐related down‐regulation of miRNAs in the *Dlk1‐Dio3* cluster could contribute to skeletal muscle atrophy.

### The *Dlk1‐Dio3* miRNA cluster regulates Atrogin‐1 protein content

**FIGURE 2:**
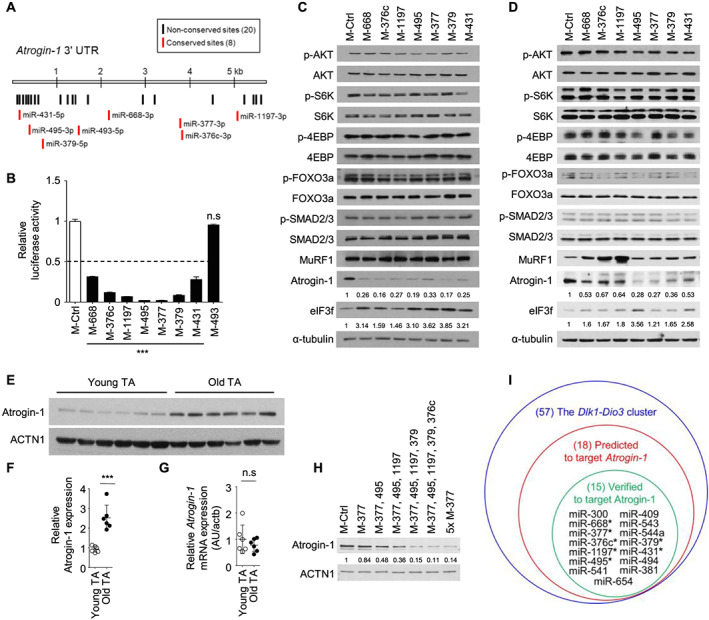
The *Dlk1‐Dio3* microRNA (miRNA) cluster inhibits Atrogin‐1 protein content in a posttranscriptional manner. (A)Twenty‐eight binding sites for miRNAs [including eight conserved sites (*red*) in humans] in the mouse *Atrogin‐1* 3′ untranslated region (UTR) were predicted. (B) Relative activity of luciferase reporters bearing the *Atrogin‐1* 3′ UTR in 293T cells transfected with the conserved miRNAs. ***P < 0.001. (C) Immunoblot analysis of the indicated proteins in differentiated C2C12 cells transfected with the indicated miRNAs. (D) Immunoblot analysis of the indicated proteins in differentiated human skeletal muscle myoblasts (HSMMs) (from a 17‐year‐old donor) transfected with the indicated miRNAs. The protein levels of Atrogin‐1 and eIF3f were normalized to α‐tubulin and quantified using ImageJ software. (E) Immunoblots of Atrogin‐1 in tibialis anterior (TA) muscles isolated from young and old mice (*n* = 6 each) and (F) quantification of Atrogin‐1. The results were normalized by the average level of ACTN1. ***P < 0.001. (G) Relative mRNA expression of *Atrogin‐1* in TA muscle isolated from young and old mice (*n* = 6). The results were normalized to those of *Actb*. (H) Immunoblots of Atrogin‐1 in differentiated C2C12 cells transfected with the indicated miRNAs. The relative abundance of Atrogin‐1 was quantified by normalization to ACTN1. (I) Schematic summary of miRNA members (*conserved in human) included in stepwise analyses.

To identify the potential targets of the miRNAs mediating the anti‐atrophic phenotype observed in mimic‐transfected myotubes, we searched for their putative binding sites using the TargetScan algorithm (www.targetscan.org). Interestingly, 18 pre‐miRNAs were predicted to bind to 28 sites on the 3′ UTR of *Atrogin‐1* encoding a muscle‐specific E3 ligase (*Figure* 2A, *Table* S1). The luciferase reporter assay was employed to further ascertain whether these miRNAs target the *Atrogin‐1* 3′ UTR. Seven out of the eight conserved pre‐miRNAs markedly reduced the luciferase reporter activity to less than half that observed with control miRNA (*Figure* 2B), while eight out of the 10 non‐conserved pre‐miRNAs did (*Figure* S1). Consistently, transfection of the seven conserved pre‐miRNAs specifically lowered Atrogin‐1 protein levels in C2C12 myotubes (*Figure* 2C). Interestingly, these miRNAs increased protein expression of eIF3f, a well‐known target of Atrogin‐1,^50^ in myotubes. These results indicated that not only the decrease in Atrogin‐1, a proteolytic enzyme, but also the increase in eIF3f, a protein translation initiation component, contributes to the anti‐atrophic effect on myotubes by miRNA mimics. In HSMMs, all conserved miRNAs inhibited Atrogin‐1 (*Figures* 2D and S2). To determine whether other anabolic or catabolic components are involved in the anti‐atrophic phenotype of miRNA mimic‐transfected myotubes, we investigated AKT, S6K, 4EBP, FOXO3a, SMAD2/3, and another E3 ligase, MuRF1. Almost all of the assayed components were not significantly affected by the overexpression of miRNAs. Although the protein status of a few components, including S6K and MuRF1, were observed to be marginally altered by the overexpression a few of the miRNAs, the Atrogin‐1 protein content was robustly down‐regulated in myotubes transfected with each miRNA mimic.

In line with the finding that miRNAs in the *Dlk1‐Dio3* cluster were significantly down‐regulated in aged muscle tissue, Atrogin‐1 protein was conversely up‐regulated in aged mouse muscle (*Figure* 2E and F). However, no significant changes in *Atrogin‐1* transcript levels were evident (*Figure* 2G), supporting the idea that the age‐related increase in Atrogin‐1 protein might be attributable to posttranscriptional regulation by the miRNAs in the cluster. To investigate whether these miRNAs could have additive effects on the inhibition of Atrogin‐1 translation, we analysed the combined effects of the top five miRNAs, which are conserved between the human and mouse genomes, as shown in *Figure* 2B, on Atrogin‐1 protein levels (*Figure* 2H). Of note, five mimics of such miRNAs had additive effects on reducing Atrogin‐1 protein content. Collectively, we demonstrated that a group of miRNAs in the *Dlk1‐Dio3* cluster control Atrogin‐1 protein content, as summarized in *Figure* 2I. These results suggested that Atrogin‐1 up‐regulation caused by collective down‐regulation of miRNAs in *Dlk1‐Dio3* with age might be an important intrinsic cue contributing to sarcopenia.

### MiR‐376c‐3p in the cluster improves muscle atrophy *in vitro*

**FIGURE 3:**
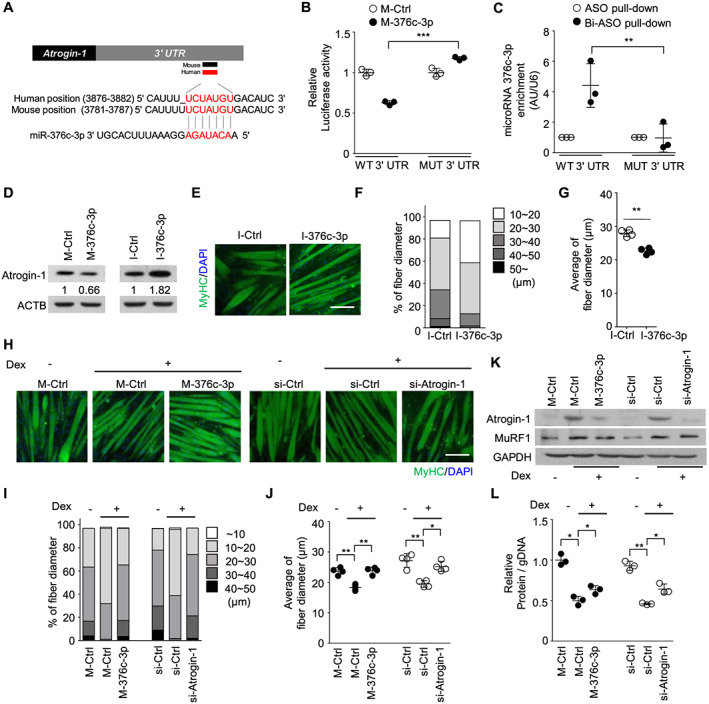
Overexpression of miR‐376c‐3p improves myotube atrophy *in vitro*. (A) The miR‐376c‐3p binding site in the mouse *Atrogin‐1* 3′ untranslated region (UTR) (positions 3781–3787) is conserved in the human *Atrogin‐1* 3′ UTR (positions 3876–3882). (B) Effects of miR‐376c‐3p on the activity of luciferase reporters bearing wild‐type (WT) or deletion mutant (Mut) of its binding site for *Atrogin‐1* 3′ UTR. ***P < 0.001. (C) C2C12 cells were transfected with the indicated firefly luciferase reporters containing WT *Atrogin‐1* 3′ UTR or mutant *Atrogin‐1* 3′ UTR with seed mutation. Forty‐eight hours after transfection, *Luciferase2* mRNA was pulled down by antisense oligonucleotide (ASO) (with or without biotin) using streptavidin beads and analysed by quantitative reverse transcription–PCR (qRT–PCR) for miR‐376c. **P < 0.01. (D) Immunoblot of Atrogin‐1 in differentiated primary myoblasts transfected with M‐miR‐376c‐3p or I‐miR‐376c‐3p. The protein levels of Atrogin‐1 were quantified using ImageJ software and normalized by ACTB. (E–G) Representative images (E), quantification graphs for a percentage (F), and average (G) of differentiated myotubes transfected with I‐miR‐376c‐3p or control. *Green*, MyHC; *blue*, 4′,6‐diamidino‐2‐phenylindole (DAPI). Scale bar, 50 μm. **P < 0.01. (H) Representative images of M‐miR‐376c‐3p or si‐Atrogin‐1‐transfected C2C12 myotubes treated with or without 100 μM of dexamethasone (Dex) for 24 h. *Green*, MyHC; *blue*, DAPI. Scale bar, 50 μm. (I, J) Quantification graphs for percentages (I) and averages (J) of fibre diameters in H. Four different views were randomly selected for measurement of myotube diameters. *P < 0.05, **P < 0.01. (K) Immunoblot analysis of Atrogin‐1 and MuRF1. Protein levels were quantified using ImageJ software and normalized by GAPDH. (L) Relative ratio of protein accumulation normalized by the genomic DNA content in M‐miR‐376c‐3p or si‐Atrogin‐1‐transfected C2C12 myotubes treated with or without 100 μM of Dex for 24 h. *P < 0.05, **P < 0.01.

To investigate the therapeutic potential of *Dlk1‐Dio3* clustered miRNAs in overcoming age‐related phenotypes *in vivo*, we selected one of the most effective miRNAs in inducing thickening of the myotube. Among the top five candidates that strongly increased myotube diameter *in vitro* (*Figure* 1C), miR‐376c‐3p was inhibited most robustly in aged TA muscle (*Figure* S3). To further examine the specific interactions between miR‐376c‐3p and *Atrogin‐1* 3′ UTR, we initially performed reporter assays using a luciferase‐*Atrogin‐1* 3′ UTR construct and a miR‐376c‐3p mimic. The mimic of miR‐376c‐3p (M‐miR‐376c‐3p) reduced luciferase activity, which was effectively abolished by the mutation in the miR‐376c‐3p binding site on the 3′ UTR (*Figure* 3A and B). Pull‐down experiments performed using biotinylated Atrogin‐1 antisense oligomers (Bi‐ASO) as bait demonstrated the direct binding between endogenous miR‐376c‐3p and the *Atrogin‐1* 3′ UTR (*Figures* 3C and S4). M‐miR‐376c‐3p transfection led to reduced Atrogin‐1 in primary myoblasts (*Figure* 3D), C2C12 and HSMMs (*Figure* S5A and S5B), while an inhibitor, (I)‐miR‐376c‐3p, increased Atrogin‐1 levels. The total protein content in cell lysates was significantly increased in M‐miR‐376c‐3p‐transfected HSMMs (*Figure* S5C). Contrary to the antiatrophy phenotype shown in M‐miR‐376c‐3p‐overexpressing myotubes, the myotubes transfected with I‐miR‐376c‐3p displayed loss of fibre thickness (*Figure* 3E–G).

Because Atrogin‐1 is a well‐known factor that causes atrophy in glucocorticoid‐treated muscle,^29^, ^51^, ^52^, ^53^, ^54^, ^55^ we investigated whether miR‐376c‐3p could improve glucocorticoid‐induced muscle atrophy *in vitro*. Interestingly, M‐miR‐376c‐3p prevented myotube atrophy induced by dexamethasone, resulting in similar fibre diameters relative to control myotubes without dexamethasone treatment (*Figure* 3H–J), along with decreased Atrogin‐1 protein content (*Figure* 3K). In addition, M‐miR‐376c‐3p treatment led to recovery, in part, of the decreased protein content in dexamethasone‐treated myotubes (*Figure* 3L). Knockdown of *Atrogin‐1* using siRNA prevented morphological deterioration and decline in protein content in dexamethasone‐treated myotubes. Our findings clearly indicate that exogenous miR‐376c‐3p can effectively attenuate muscle atrophy by targeting Atrogin‐1.

### Intramuscular delivery of miR‐376c‐3p ameliorates age‐related muscle atrophy

**FIGURE 4:**
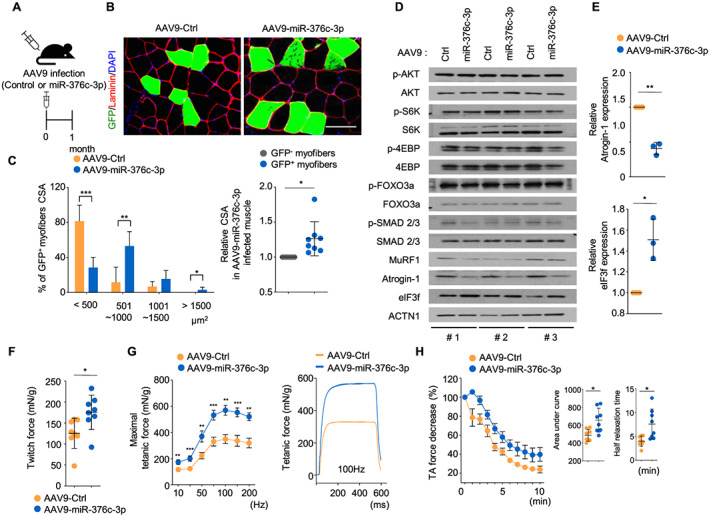
Muscle‐directed AAV9‐miR‐376c‐3p delivery ameliorates muscle atrophy in aged mice. (A) Scheme of AAV9 injections into tibialis anterior (TA) muscle (AAV9‐miR‐376c‐3p) and contralateral TA muscle (AAV9‐Ctrl) of 23‐month‐old mice. (B, C) Representative images (B) and graphs (C) for a distribution (*left*) and relative value (*right*) of cross‐sectional area in AAV9‐infected muscle. *Green*, GFP; *red*, laminin; *blue*, 4′,6‐diamidino‐2‐phenylindole (DAPI). Scale bars, 50 μm. *P < 0.05, **P < 0.01, ***P < 0.001. (D) Immunoblots of the indicated proteins in AAV9‐Ctrl or miR‐376c‐3p‐infected TA muscle tissues of 23‐month‐old mice (*n* = 3). (E) Relative abundance of Atrogin‐1 and eIF3f in immunoblots using ImageJ. The results were normalized by the average level of ACTN1. The data are presented as the mean ± SD. *P < 0.05, **P < 0.01. (F) Twitch and (G) tetanic forces, and (H) tibialis fatigue index in AAV9‐infected old mice (*n* = 8 each). Isometric forces (mN) were determined using an electrical stimulator at 1 Hz and 100 V. For analysis of resistance to muscle fatigue, the muscle was repeatedly stimulated every 30 s for 10 min. Then, the isometric forces were analysed as a percentage of the initial maximal contractile force. The data are presented as the mean ± SD. *P < 0.05.

Compared with those of 3‐month‐old mice, muscle tissues of 24‐month‐old mice exhibited sarcopenic phenotypes, with significant loss of muscle mass and smaller cross‐sectional areas (*Figure* S6). To establish whether miR‐376c‐3p improves age‐related muscle atrophy *in vivo*, TA muscles of 23‐month‐old mice were infected with AAV serotype 9 expressing GFP (AAV9‐GFP) bearing miR‐376c‐3p (AAV9‐miR‐376c‐3p) or nontarget miRNA (AAV9‐Ctrl), and then the histological and functional analyses were performed at 4 weeks after infection (*Figure* 4A). The overall tissue architecture of AA9‐miR‐376c‐3p‐infected muscle was similar to that of the contralateral muscle, showing no significant change in percentages of fibres with central nuclei (*Figure* S7). Notably, GFP‐positive miR‐376c‐3p‐overexpressing TA muscle displayed markedly larger fibres relative to contralateral TA muscle (*Figure* 4B and C). Consistent with the *in vitro* data, miR‐376c‐3p specifically inhibited Atrogin‐1 protein expression, while the levels of other components were not significantly changed. As expected, eIF3f levels were higher in the AAV9‐miRa‐376c‐3p‐infected mice than in contralateral TA muscle (*Figure* 4D and E), showing a negative correlation with Atrogin‐1 protein content. These results suggest that anti‐atrophic effects on myofibers could have resulted from the inhibition of Atrogin‐1 translation but not the alteration of anabolic resistance. To investigate whether enlarged TA muscle that was overexpressing miR‐376c‐3p showed improved muscle function, we measured isometric forces *ex vivo* using isolated TA muscle tissues treated by the same viral infection in *Figure* 4A. A previous report showed that aged muscle exhibited a lower maximal force and fatigued more rapidly than young muscle.^56^ Importantly, miR‐376c‐3p‐overexpressing muscle was significantly stronger than contralateral muscle infected with AAV9‐Ctrl, showing increased twitch and tetanic force (*Figure* 4F and G). In addition, miR‐376c‐3p ameliorated fatigue resistance in old muscle, with an approximately two‐fold increase observed in the half‐relaxation time (*Figure* 4H). Taken together, our results suggest that miR‐376c‐3p presents a valuable therapeutic target to combat muscle aging.

Atrogin‐1 serves as a high‐fidelity marker of acute muscle atrophy and is up‐regulated in multiple settings of cachexia.^57^ We additionally examined whether miR‐376c‐3p improves muscle wasting resulting from tumour‐induced atrophy. Consistent with the previous reports,^58^ C2C12 myotubes in colon‐26 (C26) conditioned medium exhibited markedly thinner fibre diameters than those in normal medium. Notably, M‐miR‐376c‐3p transfection led to the recovery of the diameter of myotubes in C26 conditioned medium to a similar extent as control myotubes in normal medium (*Figure* S8). To ascertain the therapeutic potential of miR‐376c‐3p in a cachexia mouse model bearing C26 tumours, we injected TA muscle with either AAV9‐Ctrl or AAV9‐miR‐376c‐3p, inoculated C26 tumour cells at 1 week after infection, and sacrificed mice on Day 21 (*Figure* S9A). The body weights of tumour‐bearing mice were slightly but significantly lower than those of non‐tumour mice. TA muscle weights normalized by tibia length were decreased in tumour‐bearing mice (*Figure* S9B). TA muscle infected with AAV9‐miR‐376c‐3p showed 16% weight loss whereas 22% weight loss was observed in contralateral TA muscle infected with AAV9‐Ctrl (*Figure* S9C). The decreased weight loss in TA muscle infected with AAV9‐miR‐376c‐3p was accompanied by an increase in cross‐sectional area (*Figure* S9D). Consistently, protein expression of Atrogin‐1 was inhibited in AAV9‐miR‐376c‐3p infected muscle of tumour‐bearing mice (*Figure* S9E). Our findings clearly indicate that delivery of exogenous miR‐376c‐3p can effectively attenuate muscle atrophy in tumour‐induced cachexia.

### Expression of miRNAs in the *Dlk1‐Dio3* cluster decreased with age in human muscle

**FIGURE 5:**
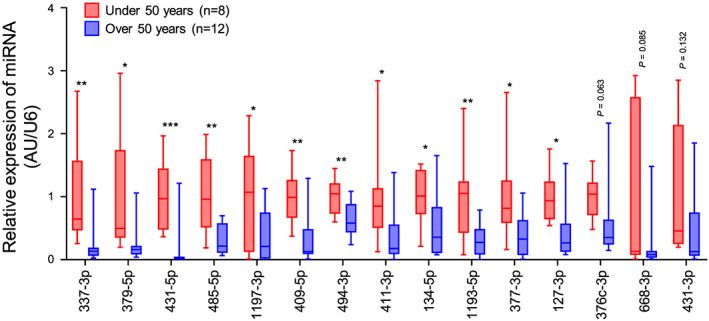
Down‐regulation of the *Dlk1‐Dio3* cluster microRNAs (miRNAs) in human muscle tissues from individuals > 50 years old. The relative expression of 15 miRNAs in the human *Dlk1‐Dio3* cluster was quantified by quantitative reverse transcription–PCR (qRT–PCR) analysis. RNA was isolated from human gluteus maximus muscle (from 25‐ to 80‐year‐old individuals). The data were normalized to the U6 snRNA level and are presented as the mean ± SD. *P < 0.05, **P < 0.01, ***P < 0.001.

On the basis of our previous findings in mice, we investigated the expression patterns of human miRNAs clustered in the locus with regard to age in human skeletal muscle tissues. The human *Dlk1‐Dio3* locus contains 99 mature miRNAs (54 pre‐miRNAs), 87 of which are conserved between the human and mouse genomes. Fifteen randomly selected conserved pre‐miRNAs from skeletal muscle tissue samples (*n* = 20) from human participants (ages ranging from 25 to 80 years) were subjected to qRT–PCR analysis. Consistent with the down‐regulation of clustered miRNAs observed in mice (*Figure* S10), 12 pre‐miRNAs showed a significant decrease in expression (*P* < 0.05), and the levels of the remaining three pre‐miRNAs showed a tendency to decrease in muscle samples from individuals > 50 years old (*Figure* 5). Despite the limited number of human samples for correlation analysis, we also observed that the expression of five miRNAs appeared to be negatively correlated with age, showing an ‘*r*’ value above 0.5 (*Figure* S11). These patterns of miRNAs in the *Dlk1‐Dio3* cluster in both mice and humans suggest an important role of this miRNA cluster in muscle aging.

## Discussion

Sarcopenia is currently a global public health issue owing to the aging population worldwide, but the underlying molecular mechanisms have yet to be established. Here, we demonstrated that the majority of miRNAs encoded within the *Dlk1‐Dio3* genomic locus decline in expression with age, and a number of these miRNAs cotarget a common transcript, *Atrogin‐1*, leading to its age‐dependent up‐regulation and consequent muscle atrophy. Among the miRNAs within the cluster, miR‐668,miR‐376c,miR‐1197,miR‐495,miR‐377,miR‐379,miR‐431, and miR‐493 are conserved between the human and mouse genomes. Our results showed that genetic intervention using one of these miRNAs, miR‐376c‐3p, leads to a significant improvement in skeletal muscle atrophy in aged mice via inhibition of Atrogin‐1. Our findings provide valuable targets to slow muscle aging.

The *Dlk1‐Dio3* locus is the largest mammal‐specific miRNA cluster where miRNAs are encoded by the antisense *Rtl1* and the larger transcript *Mirg* in the maternal allele.^19^ Our previous studies have established the idea that most miRNAs located in the *Dlk1‐Dio3* genomic region were significantly down‐regulated in aged muscle tissues or myoblasts, implying that the locus might be important in muscle aging.^15^, ^16^ In the present study, we focused on the functional relevance between miRNAs located in *Dlk1‐Dio3* and muscle atrophy, which is a major phenotype of aged muscle. Clustered miRNAs tend to be coexpressed in specific conditions, and the evolution of miRNA clusters frequently has similar seed families owing to gene duplication events.^59^, ^60^, ^61^ Recent experiments have shown that coexpressed miRNA clusters consisting of three to six members can regulate a specific molecular pathway by targeting the same signalling pathway.^13^, ^14^ The miR‐183 cluster (bearing miR‐183,miR‐96, and miR‐182) and the miR‐17‐92 cluster (bearing miR‐17,miR‐18a,miR‐19a,miR‐19b, and miR‐92a) controlled neuropathic pain by repressing auxiliary voltage‐gated calcium channel subunits and multiple voltage‐gated potassium channel subunits, respectively. Our results provide the first evidence of age‐dependent coexpression of the largest group of clustered miRNAs in the *Dlk1‐Dio3* locus (at least 15 in mice and seven in humans) to inhibit a common atrophy‐related gene target, *Atrogin‐1*.

**FIGURE 6:**
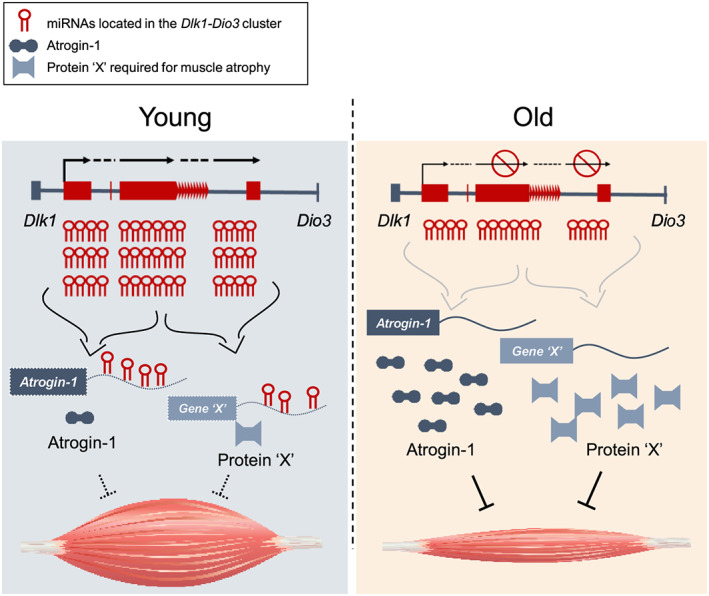
Proposed model for the age‐related regulation of Atrogin‐1 protein expression by microRNAs (miRNAs) in the *Dlk1‐Dio3* cluster. Atrogin‐1 protein levels were increased by collective down‐regulation of miRNAs in the *Dlk1‐Dio3* genomic region in older muscle tissue. Age‐related induction of Atrogin‐1 could accelerate muscle aging. This event might be an important intrinsic cue contributing to sarcopenia.

Atrogin‐1 is a muscle‐specific E3 ligase, along with MuRF1, that controls muscle homeostasis. Overexpression of Atrogin‐1 in muscle tissues caused atrophic phenotypes *in vitro*, whereas Atrogin‐1‐deficient mice have been reported to be resistant to atrophy.^29^, ^51^, ^52^ Unlike other muscular disorders, the role of Atrogin‐1 on muscle aging is relatively unknown. Importantly, our experiments showed significantly increased Atrogin‐1 protein levels in mouse older muscle tissue but no changes in the corresponding transcriptional levels, suggesting that miRNAs regulate Atrogin‐1 protein content in a posttranscriptional manner (*Figure* 6). Studies to date have reported that miR‐19a/b and miR‐23a trigger muscle hypertrophy through targeting Atrogin‐1^62^, ^63^ but are not differentially expressed between young and aged muscle tissues (*Figure* S12). In view of these collective findings, we conclude that the age‐related inhibition of miRNAs in the *Dlk1‐Dio3* cluster is an important intrinsic cue for Atrogin‐1‐mediated induction of muscle atrophy in aged muscle. Atrogin‐1 has been well known to be induced in cachexia.^57^ As expected, our genetic intervention using miR‐376c‐3p ameliorated muscle atrophy in a cancer cachexia model of colon‐26 tumour‐bearing mice by inhibition of Atrogin‐1. Although the role of miRNAs in the cluster in cachexia remains to be elucidated, our results suggest a therapeutic potential of miR‐376c‐3p in improving Atrogin‐1‐mediated muscle atrophy in cachexia.

Several studies reported transcript or protein expression of Atrogin‐1 in muscle tissues of mice, rats, rabbits, and humans with age. Most papers have described only transcript levels of *Atrogin‐1* in aged muscle even though the results are controversial, implying that sarcopenia may not be caused by an acute increase of Atrogin‐1 transcript as in the case of disuse, denervation, and cachexia. Although a few papers have shown Atrogin‐1 protein levels, the protein content with age in humans remains elusive. In the present study, we observed a simultaneous down‐regulation of the *Dlk1‐Dio3* cluster miRNAs in aged mouse and demonstrated that a subset of miRNAs in the cluster could directly regulate Atrogin‐1 expression. In line with the finding that miRNAs in the *Dlk1‐Dio3* cluster were significantly down‐regulated in aged muscle tissue, Atrogin‐1 protein was conversely up‐regulated. Consistent with our mouse data, we found conserved miRNAs in the cluster to be robustly down‐regulated in human muscle from subjects aged >50 years, and such miRNAs directly inhibited Atrogin‐1 protein expression in human myoblasts. These results suggest a recapitulative and conserved posttranscriptional control of age‐associated muscle decline in human. On the basis of our mouse data and *in vitro* human data, we carefully infer that the Atrogin‐1 protein content may be observed to increase in aged human muscle, whereas miRNAs in the cluster should be down‐regulated. Future investigations will be needed to clarify its expression in human muscle aging.

We demonstrated that the top five miRNAs, which are conserved between the human and mouse genomes, with inhibitory effects on *Atrogin‐1* 3′ UTR luciferase activity showed an additive effect for reducing the Atrogin‐1 protein contents. Because Atrogin‐1 is a well‐known factor for causing muscle atrophy, the precise control of its expression with age would be important for healthy muscle aging. It has been reported that two clusters, miR‐449 and miR‐34b/c, have a functional redundancy in murine testes and normal brain development.^64^, ^65^ Likewise, the high redundancy of miRNAs in the *Dlk1‐Dio3* locus might not be due to inefficient regulation but may be crucial for the stringent regulation of Atrogin‐1 expression to maintain muscle homeostasis.

Our next question is with regard to the regulatory mechanisms of age‐related inhibition in the expression of the clustered miRNAs located in this *Dlk1‐Dio3* locus, where multiple long and short noncoding RNAs are expressed from the maternal allele and protein coding genes, such as *Dlk1*, *Rtl1*, and *Dio3*, are expressed from the paternal allele.^66^ The status of methylation in an intergenic differentially methylated region (IG‐DMR) located between *Dlk1* and *Gtl2* controls germline imprinting in the placenta, while methylation in MEG3‐DMR located between *Gtl2* and *Dio3* maintains imprinting post fertilization, thus enabling epigenetic control of the *Dlk1‐Dio3* miRNA cluster.^67^, ^68^, ^69^ The genes and miRNAs in the *Dlk1‐Dio3* locus were epigenetically silenced during cellular reprogramming in induced pluripotent stem cells (iPSCs) by DNA methylation and histone deacetylation, while overall mRNA and miRNA expression patterns were indistinguishable from embryonic stem (ES) cells, likely resulting in incomplete reprogramming of iPSCs.^70^, ^71^ On the other hand, a transcription factor, MEF2A, appeared to have a crucial role in the regulation of the *Dlk1‐Dio3* miRNA cluster in muscle regeneration.^22^ A recent study showed that a transcription elongation factor, AFF3, could enhance the expression of the ~200 kb polycistronic transcript within the *Dlk1‐Dio3* locus on the active allele, unless it was sequestered by association with the superelongation complex (SEC) on the inactive allele, depending on the methylation status of IG‐DMR.^72^ However, with respect to aging, nothing has been proven yet in the regulation of this locus. Thus, future studies will be needed to address whether the repression of *Dlk1‐Dio3*‐located miRNAs in human and mouse muscle is ruled by an epigenetic programme or any other master regulator.

In conclusion, we found that the conserved miR‐376c‐3p, one of the most effective miRNAs for myotube thickening, regulates Atrogin‐1 protein content in skeletal muscle, ameliorates skeletal muscle atrophy, and improves muscle function in old mice. Taking our findings together, we propose that miRNAs, including miR‐376c‐3p, targeting *Atrogin‐1* 3′ UTR would be valuable candidates for the development of therapies for maintaining muscle homeostasis during aging.

## Author Contributions

K.‐P.L. and K.‐S.K. designed and supervised the study. Y.J.S., Y.H.S., and E.‐S.K. performed the experiments and analysed the data. S.‐M.L., S.‐K.K., J.Y.K., and Y.S. performed genomic analyses. K.‐W.M. and J.‐H.Y. conducted the miRNA pull‐down experiment. B.L., Y.‐S.K., and J.S.K. contributed to *ex vivo* muscle functional analysis. J.Y.C., Y.R.Y., and S.K. supported the animal study. J.‐Y.L. and H.C.J. provided human samples and clinical data. Y.J.S., Y.S., K.‐P.L., and K.‐S.K. wrote the manuscript.

## Conflict of interest

Y.J.S., E.‐S.K., S.‐M.L., S.‐K.K., K.‐W.M., J.‐Y.L., B.L., J.S.K., J.Y.K., Y.H.S., J.Y.C., Y.R.Y., S.K., Y.‐S.K., H.C.J., Y.S., J.‐H.Y., K.‐P.L., and K.‐S.K. declare that they have no conflict of interest.

## Supporting information


**Figure S1.** **Relative luciferase activity of non‐conserved miRNAs.** Relative activity of luciferase reporters bearing the *Atrogin‐1* 3′ UTR in 293 T cells transfected with the indicated miRNAs. The data are presented as the mean ± SD. *P < 0.05, **P < 0.01, ***P < 0.001.
**Figure S2. Immunoblot analysis of anabolic and catabolic components in differentiated HSMMs (from a 19‐year‐old donor).** Immunoblots of the indicated proteins from differentiated HSMMs (from a 19‐year‐old donor) transfected with the indicated miRNAs. The Atrogin‐1 and eIF3f protein levels were quantified using ImageJ and normalized to α‐tubulin.
**Figure S3. Expression levels of the top 5 miRNAs inducing the antiatrophic phenotype on C2C12 myotubes in young and aged TA muscle.** Relative expression of miR‐668, 376c, 494, 541, and 1197 in young and aged TA muscle (*n* = 5). The data were normalized to the U6 snRNA level and presented as the mean ± SD (*P < 0.05).
**Figure S4. Pull‐down of *Luciferase 2* containing wild‐type (WT) or deletion mutant (Mut) miR‐376c‐3p binding site in *Atrogin‐1* 3′ UTR.** C2C12 cells were transfected with the indicated luciferase reporters containing wild‐type (WT) or deletion mutant (Mut) miR‐376c‐3p binding sites in the *Atrogin‐1* 3′ UTR. At 48 h after transfection, *Luciferase 2* mRNA was pulled down using ASO (in the presence or absence of biotin) with streptavidin beads, and RT‐qPCR analysis was performed to detect *Luciferase 2* mRNA enrichment. The data are presented as the mean ± SD of 3 independent experiments.
**Figure S5. miR‐376c‐3p inhibits Atrogin‐1 protein content in fully differentiated C2C12 cells and HSMMs.** (A, B) Immunoblot analysis of Atrogin‐1 in M‐miR‐376c‐3p‐ and I‐miR‐376c‐3p‐transfected C2C12 cells (A) and HSMMs (B). The results were normalized to ACTB levels. (C) Ratio of protein accumulation normalized by genomic DNA content in differentiated HSMMs transfected with M‐miR‐376c‐3p or control. The data are presented as the mean ± SD. **P < 0.01.
**Figure S6. Aged mice exhibit loss of muscle mass.** (A) Ratio of muscle weights to body weight. Hind limb muscles were isolated from 3‐ and 24‐month‐old mice. (B) *(left)* Representative images of cross sections of young and old TA muscle. *n* = 4, each group. *Red*, laminin; *Blue*, DAPI. Scale bar, 50 μm. *(right)* Morphometric analysis of cross‐section areas (CSAs). Six different views were randomly selected, and each CSA was measured using ImageJ software (*P < 0.05, **P < 0.01).
**Figure S7. Histological analysis of AAV9 infected TA muscle of 23‐month‐old mice.** Representative H&E images (*left*) and a quantification graph (*right*) showing the percentages of regenerating fibre with central nuclei (***black arrow***). Scale bar, 50 μm.
**Figure S8. Overexpression of miR‐376c‐3p improves muscle atrophy induced by C26 cultured media.** Representative images (A), quantification graphs for a percentage (B) and average (C) of fibre diameters, immunoblots of Atrogin‐1 and eIF3f (D) in M‐miR‐376c‐3p or Ctrl‐transfected C2C12 myotubes incubated with or without colon‐26 (C26) cultured medium (CM). The protein levels of Atrogin‐1 and eIF3f were normalized to α‐tubulin and quantified using ImageJ. The data are presented as the mean ± SD, *n* = 5. *P < 0.05.
**Figure S9. miR‐376c‐3p ameliorates muscle atrophy in a cancer cachexia model of C26 tumour‐bearing mice.** (A) Scheme of AAV9 injections into TA muscle (AAV9‐miR‐376c‐3p) and contralateral TA muscle (AAV9‐Ctrl) of C26 tumour‐bearing mice (*n* = 14). (B) (*left*) Body weights and (*right*) ratio of TA muscle weight to tibia length were measured before and after colon 26 tumour inoculation at 21 days. (C) Percentage of changed weight of TA muscle tissues infected with AAV9‐miR‐376c‐3p or control virus at 21 days post‐tumour injection. (D) Morphometric analysis of a cross‐section area (CSA). (E) Immunoblot analysis of Atrogin‐1 in AAV9 infected muscle of tumour‐bearing mice. The relative abundance of Atrogin‐1 was quantified by normalization to ACTN1. The data are presented as mean ± SD (*P < 0.05, **P < 0.01, ***P < 0.001)*.*
**Figure S10. Comparative analysis of miRNA expression profiles in TA muscles and myoblasts isolated from young and aged mice.** (A) The charts display differentially expressed miRNAs in TA muscle tissues with age. (*left*) The circle chart depicts that 58% of miRNAs are downregulated in aged muscle TA. (*right*) The pie chart shows that 68% of miRNAs are located in the *Dlk‐Dio3* genomic region. (B) Unsupervised hierarchical clustering of 42 miRNAs located in the *Dlk‐Dio3* genomic region based on aging. All miRNAs were downregulated. Each column represents miRNA levels in young (6‐month‐old) and aged (24‐month‐old) (*n* = 5) TA muscles. (C) The charts display differentially expressed miRNAs in myoblasts isolated from young (3‐month‐old) and old (27‐month‐old) mice. (*left*) The circle chart depicts that 60% of miRNAs are downregulated in old myoblasts. (*right*) Among these, the pie chart shows that 83% of the miRNAs are located in the *Dlk‐Dio3* genomic region. (D) Unsupervised hierarchical clustering of 59 miRNAs located in the *Dlk‐Dio3* genomic region with aging. All miRNAs were downregulated. Each column represents miRNA levels in myoblasts isolated from young and old TA muscles (*n* = 3). The intensity represents the magnitude of the difference. Red and green denote high and low expression, respectively.
**Figure S11. Correlation analysis of 5 miRNAs located in the human *Dlk1‐Dio3* cluster.** Expression of miRNAs between humans of different ages (25 to 80 years) was quantified via qRT‐PCR. RNA was isolated from gluteus maximus muscle. The data were evaluated using Spearman's correlation test (ρ; 95% CI; *n* = 20).
**Figure S12. Expression levels of miR‐23a, 19a, and 19b in young and old TA muscle.** (A) Correlation analysis between human age and expression of miR‐23a‐3p quantified via qRT‐PCR. Data were evaluated using Spearman's correlation test (ρ; 95% CI). (B) Relative expression of miR‐23a, 19a, and 19b in young and aged TA muscles (*n* = 5).
**Table S1. Summary of miRNAs predicted to interact with the mouse *Atrogin‐1* 3′ UTR. Twenty‐eight** binding regions were predicted to interact with the mouse *Atrogin‐1* 3′ UTR. *Conserved sites in human *Atrogin‐1* 3′ UTR.
**Table S2.** List of miRNA mimics (Ambion)
**Table S3.** List of miRNA mimics (Bioneer)
**Table S4.** List of siRNA oligos (Bioneer)
**Table S5.** Primer sequences for RT‐qPCR
**Table S6.** List of Taqman probes for the detection of miRNA expression (Ambion)
**Table S7.** Primers used in ASO pull‐down analysis
