# Supplementary material for: A subset of microRNAs in the Dlk1‐Dio3 cluster regulates age‐associated muscle atrophy by targeting Atrogin‐1
Source: J Cachexia Sarcopenia Muscle. 2020 Jun 3;11(5):1336–50. doi: 10.1002/jcsm.12578 (PMC7567143; doi:10.1002/jcsm.12578)

Fig. 2C

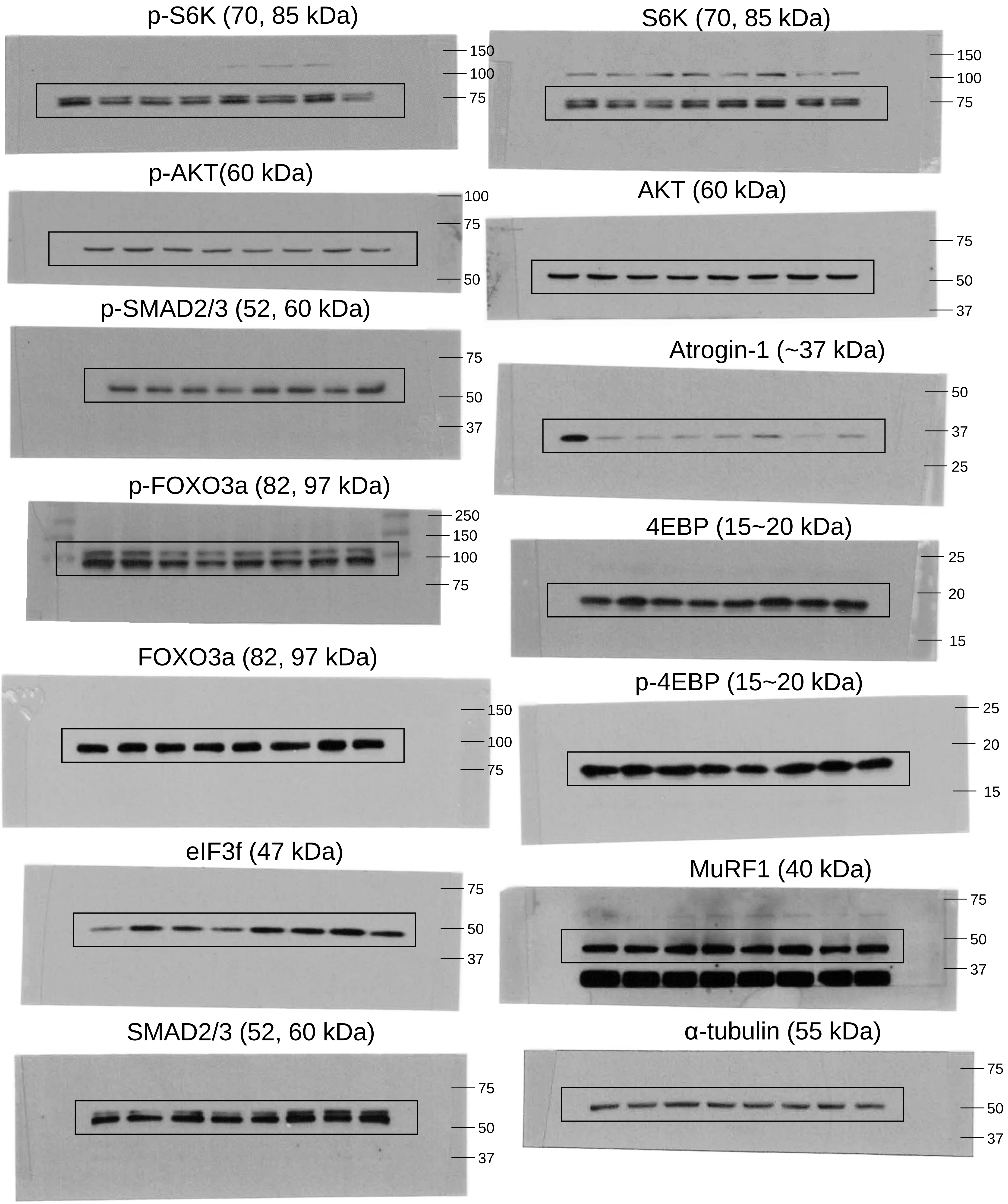
p-S6K (70, 85 kDa)
150
100
75
S6K (70, 85 kDa)
150
100
75
p-AKT(60 kDa)
100
75
50
AKT (60 kDa)
75
50
37
p-SMAD2/3 (52, 60 kDa)
75
50
37
Atrogin-1 (~37 kDa)
50
37
25
p-FOXO3a (82, 97 kDa)
250
150
100
75
4EBP (15~20 kDa)
25
20
15
FOXO3a (82, 97 kDa)
150
100
75
p-4EBP (15~20 kDa)
25
20
15
eIF3f (47 kDa)
75
50
37
MuRF1 (40 kDa)
75
50
37
SMAD2/3 (52, 60 kDa)
75
50
37
α-tubulin (55 kDa)
75
50
37

Fig. 2D

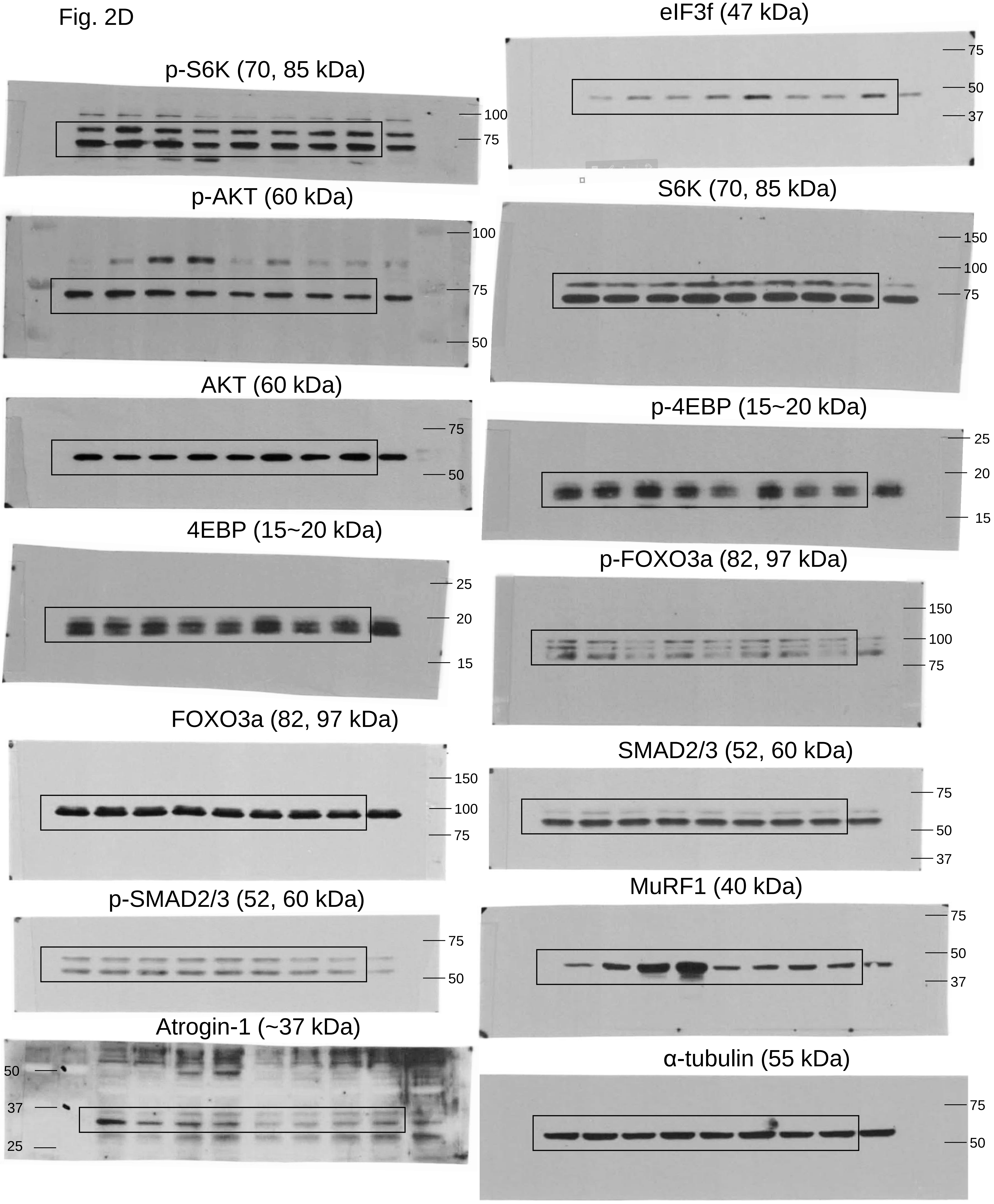

p-S6K (70, 85 kDa)
100
75
p-AKT (60 kDa)
100
75
50
AKT (60 kDa)
75
50
4EBP (15~20 kDa)
25
20
15
FOXO3a (82, 97 kDa)
150
100
75
p-SMAD2/3 (52, 60 kDa)
75
50
Atrogin-1 (~37 kDa)
50
37
25
eIF3f (47 kDa)
75
50
37
S6K (70, 85 kDa)
150
100
75
p-4EBP (15~20 kDa)
25
20
15
p-FOXO3a (82, 97 kDa)
150
100
75
SMAD2/3 (52, 60 kDa)
75
50
37
MuRF1 (40 kDa)
75
50
37
α-tubulin (55 kDa)
75
50

Fig. 2E

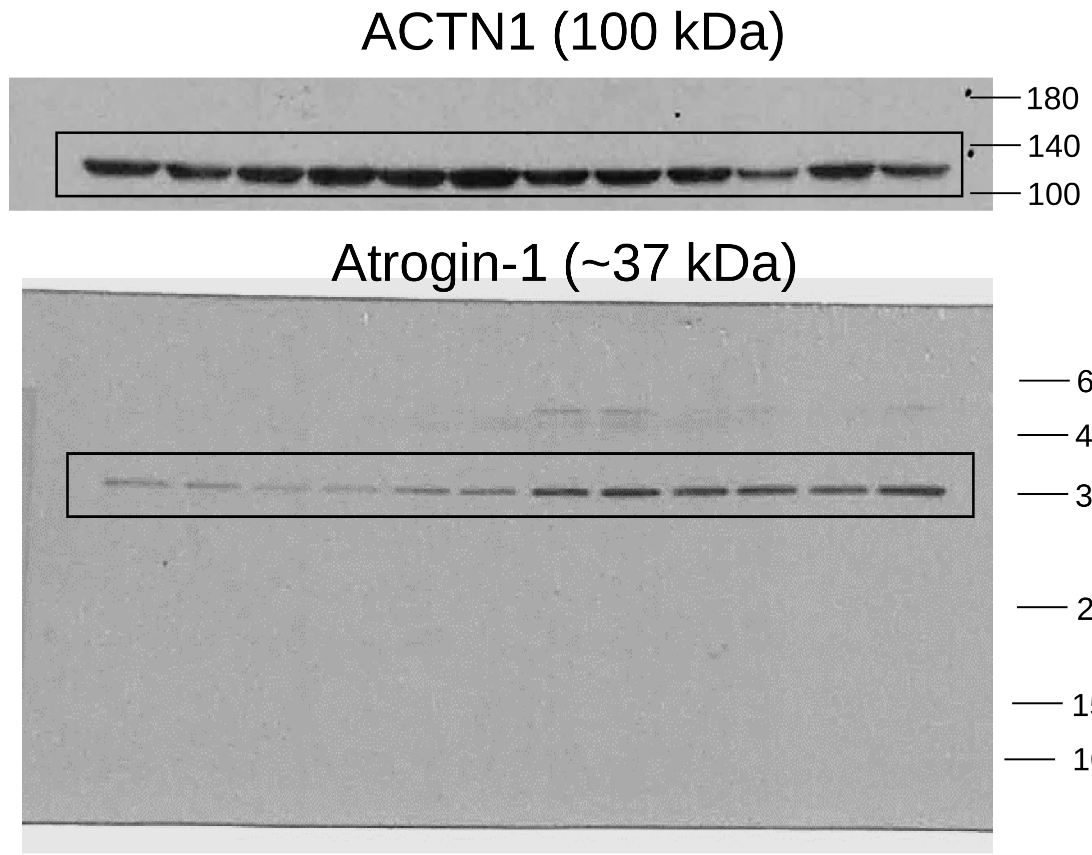

Fig. 2H

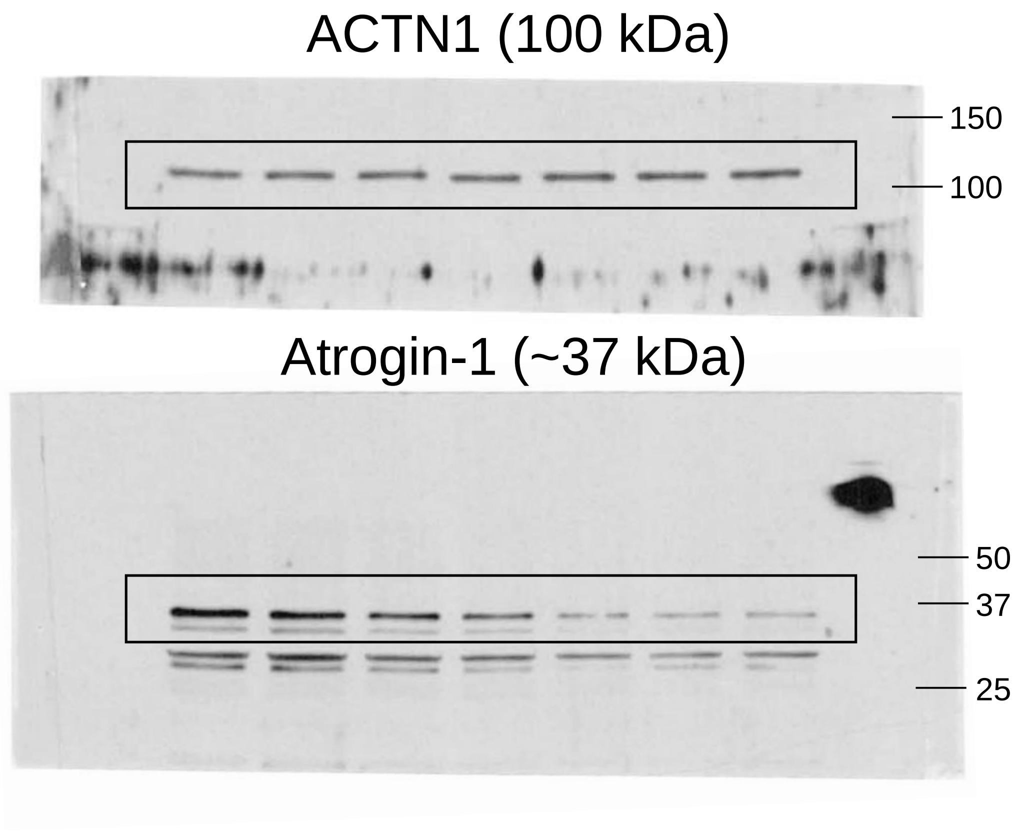

Fig. 3D

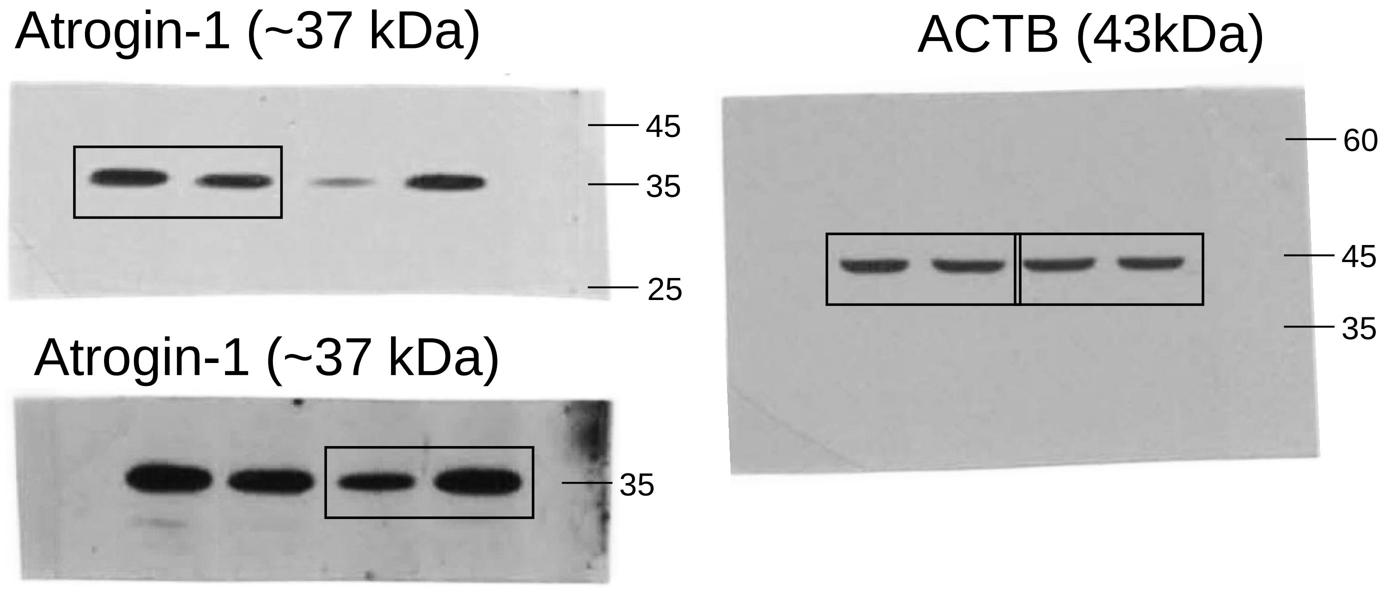

Fig. 3K

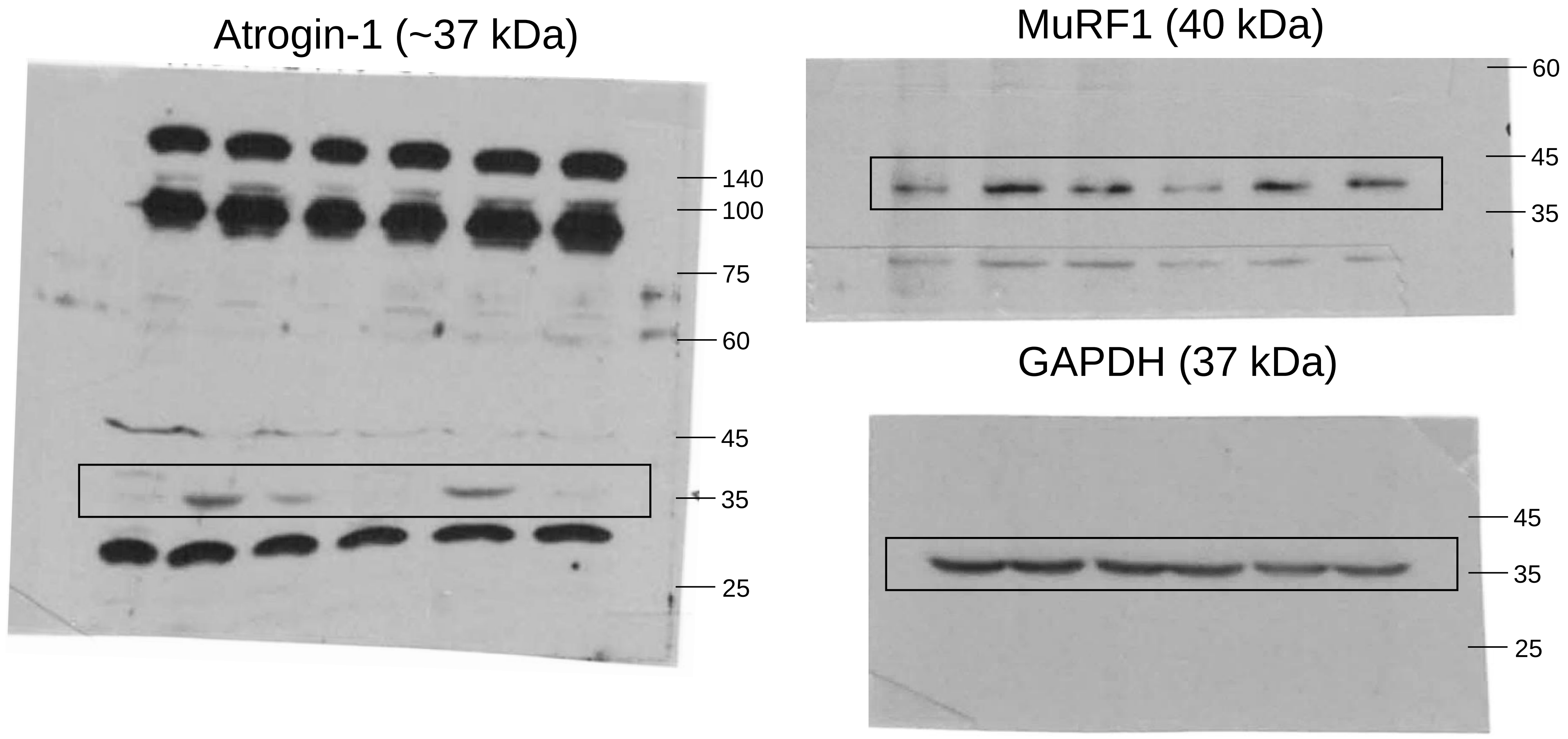

Fig. 4D

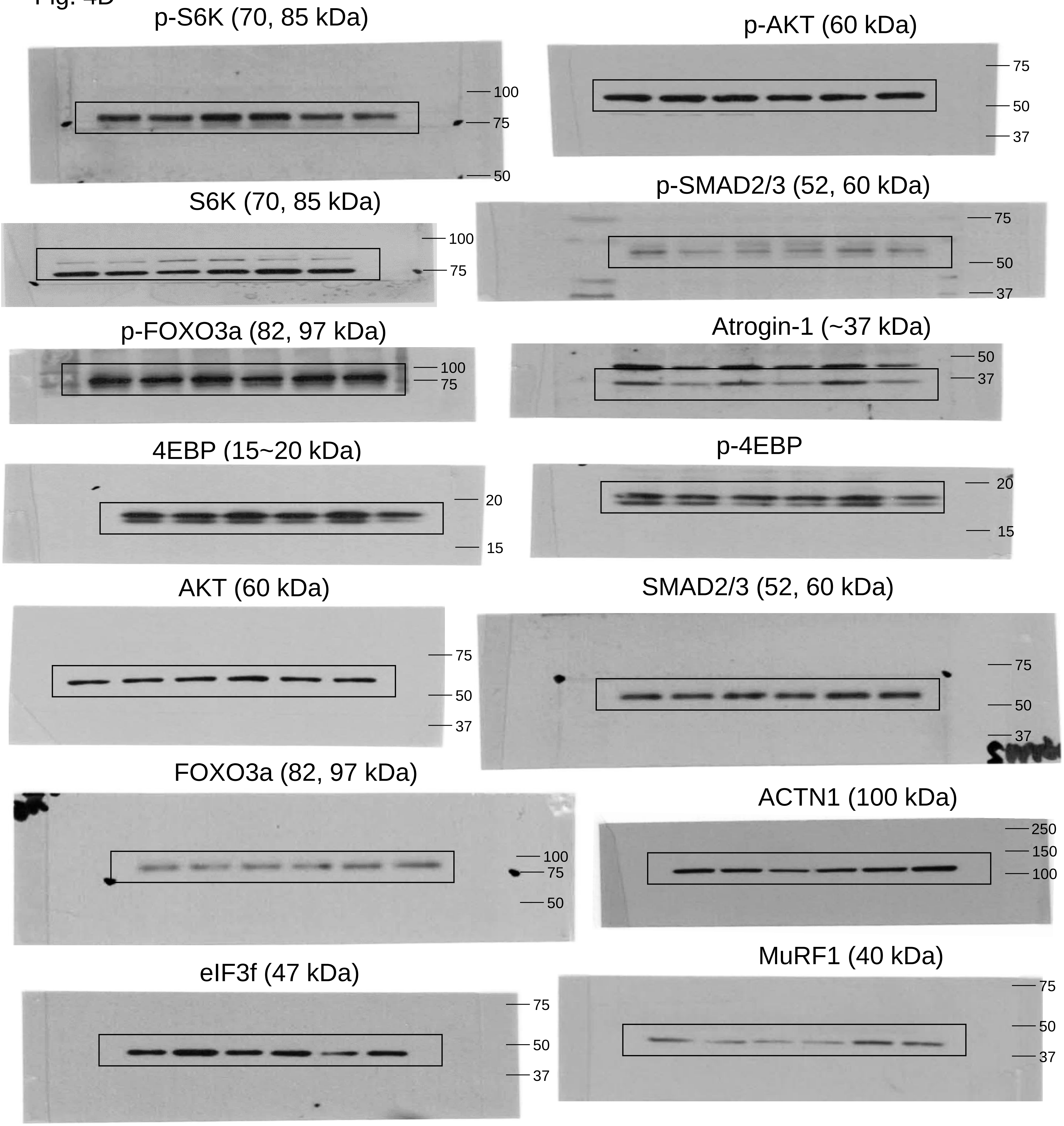

p-S6K (70, 85 kDa)
100
75
50
p-AKT (60 kDa)
75
50
37
S6K (70, 85 kDa)
100
75
p-SMAD2/3 (52, 60 kDa)
75
50
37
p-FOXO3a (82, 97 kDa)
100
75
Atrogin-1 (~37 kDa)
50
37
4EBP (15~20 kDa)
20
15
p-4EBP
20
15
AKT (60 kDa)
75
50
37
SMAD2/3 (52, 60 kDa)
75
50
37
FOXO3a (82, 97 kDa)
100
75
50
ACTN1 (100 kDa)
250
150
100
eIF3f (47 kDa)
75
50
37
MuRF1 (40 kDa)
75
50
37

Fig. S2

p-S6K (70, 85 kDa)

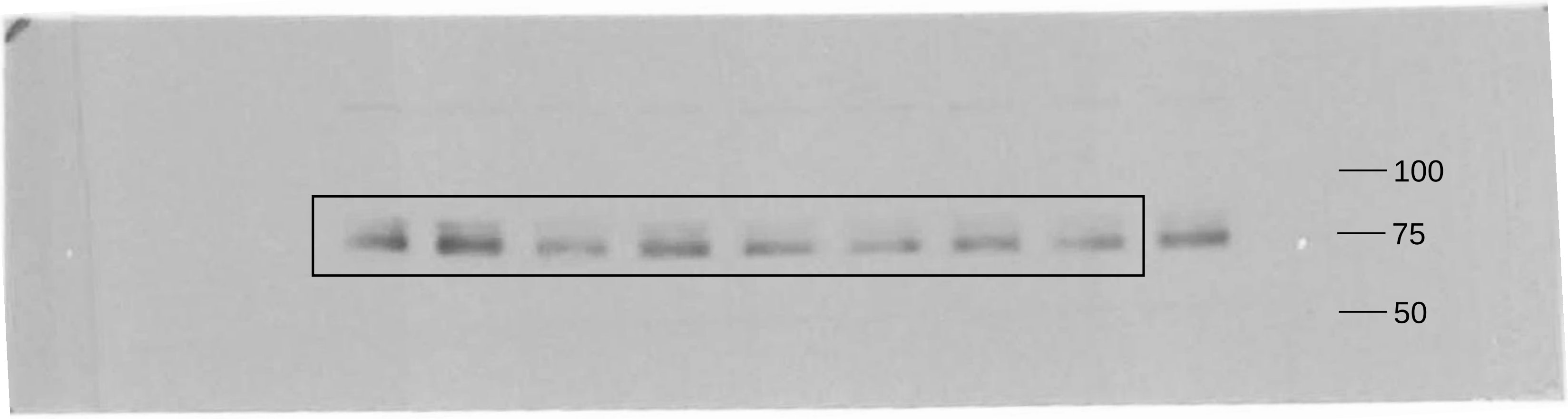

p-AKT (60 kDa)

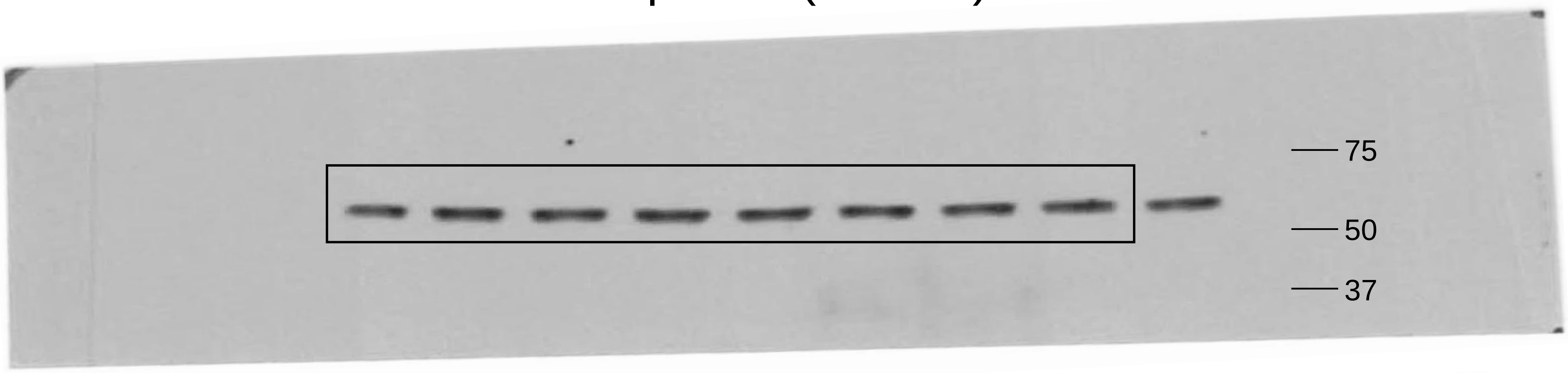

S6K (70, 85 kDa)

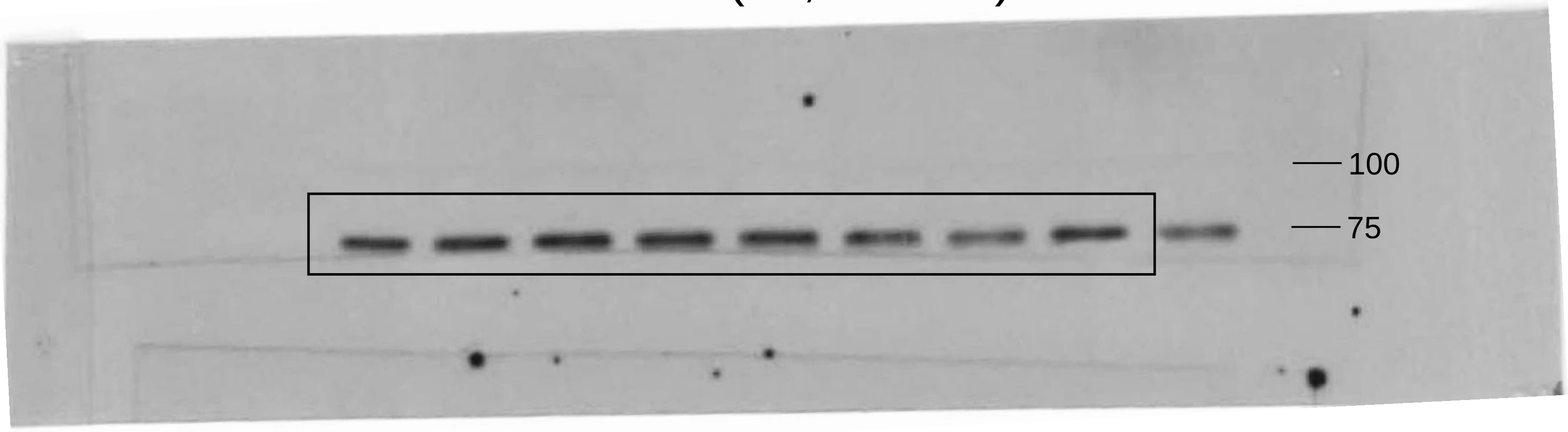

p-FOXO3a (82, 97 kDa)

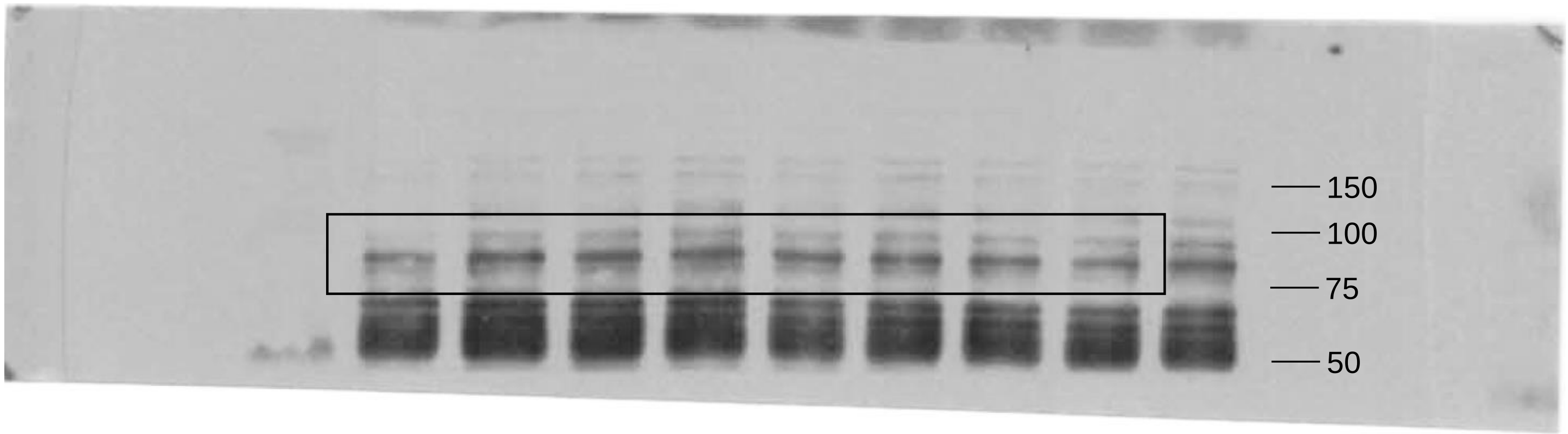

Atrogin-1 (~37 kDa)

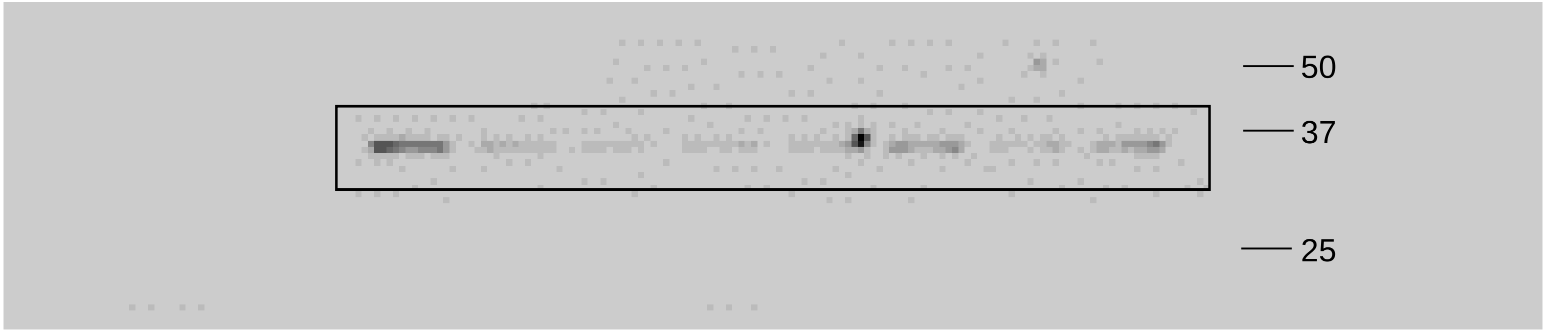

Fig. S2

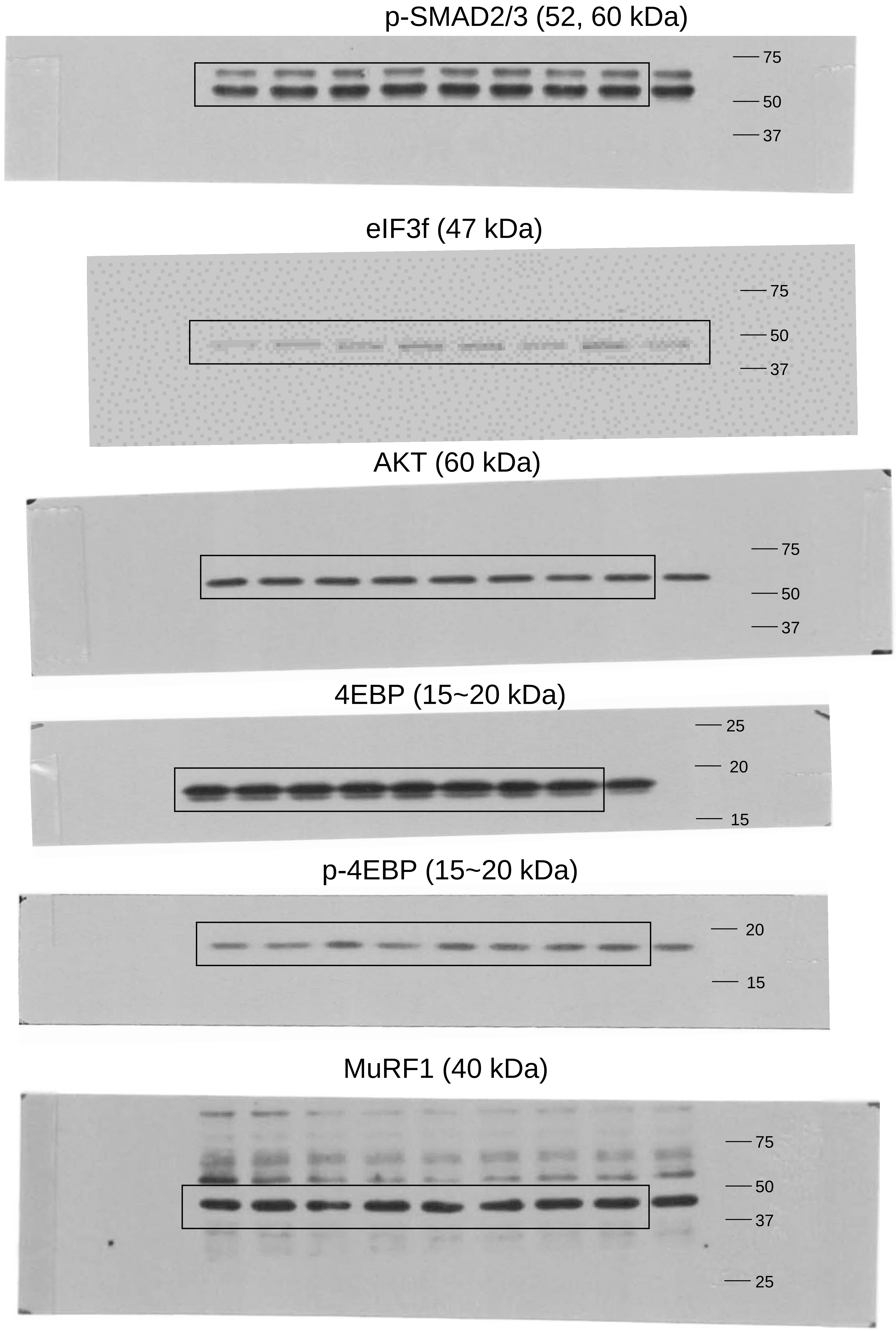

p-SMAD2/3 (52, 60 kDa)
75
50
37
eIF3f (47 kDa)
75
50
37
AKT (60 kDa)
75
50
37
4EBP (15~20 kDa)
25
20
15
p-4EBP (15~20 kDa)
20
15
MuRF1 (40 kDa)
75
50
37
25

Fig. S2

FOXO3a (82, 97 kDa)

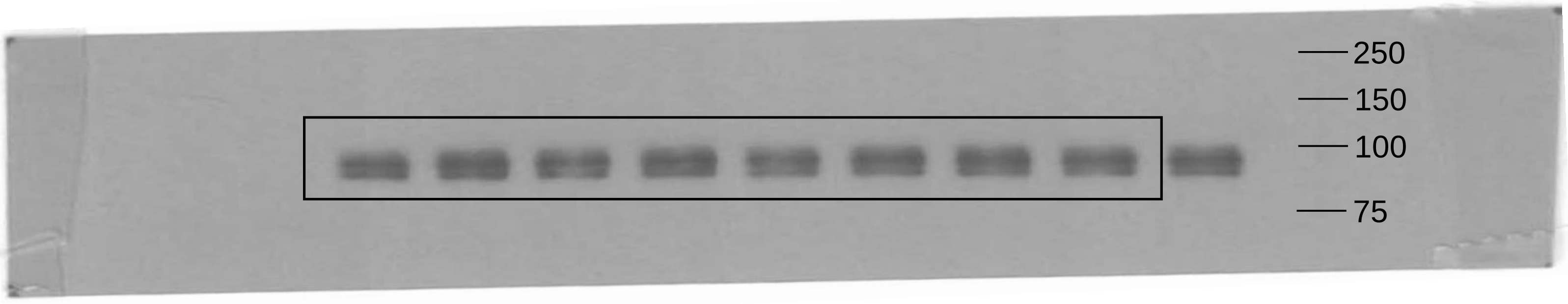

SMAD2/3 (52, 60 kDa)

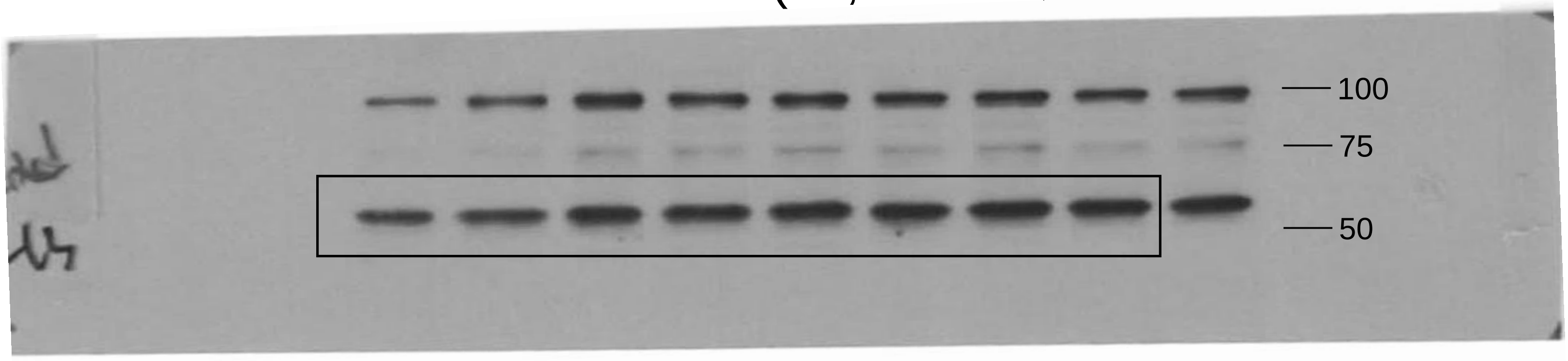

α-TUBULIN (55 kDa)

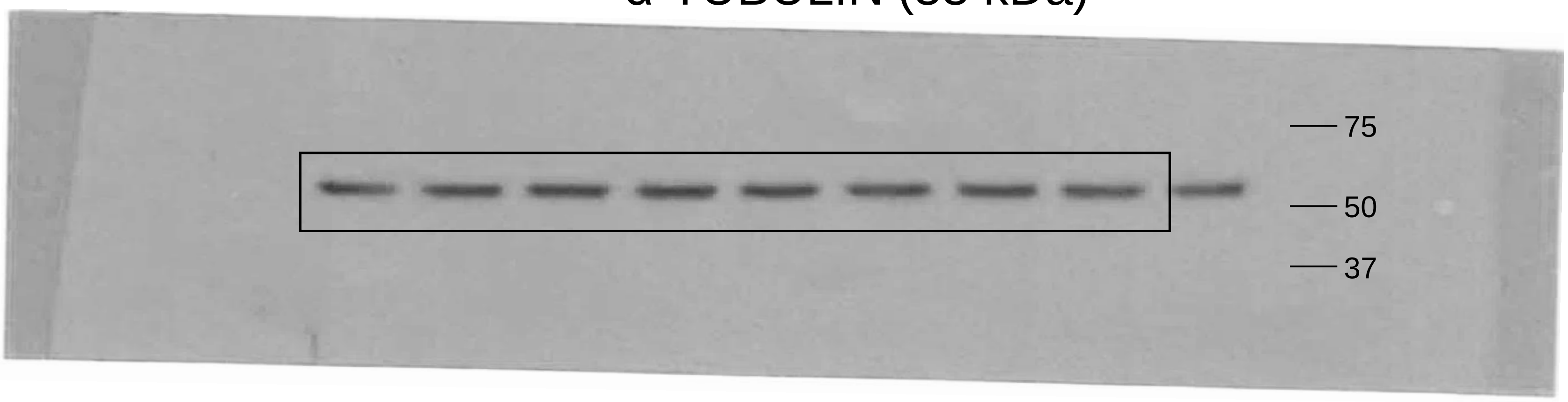

Fig. S5A

Atrogin-1 (~37 kDa)

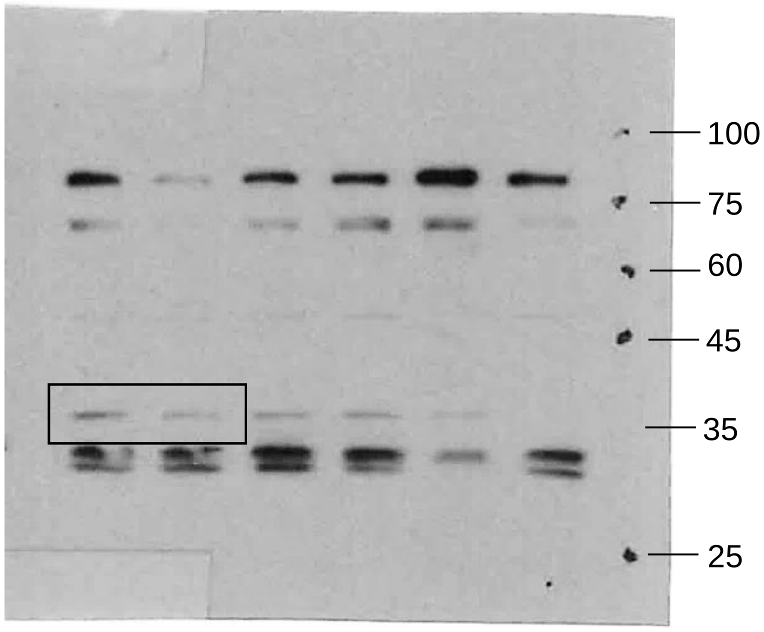

ACTB (43 kDa)

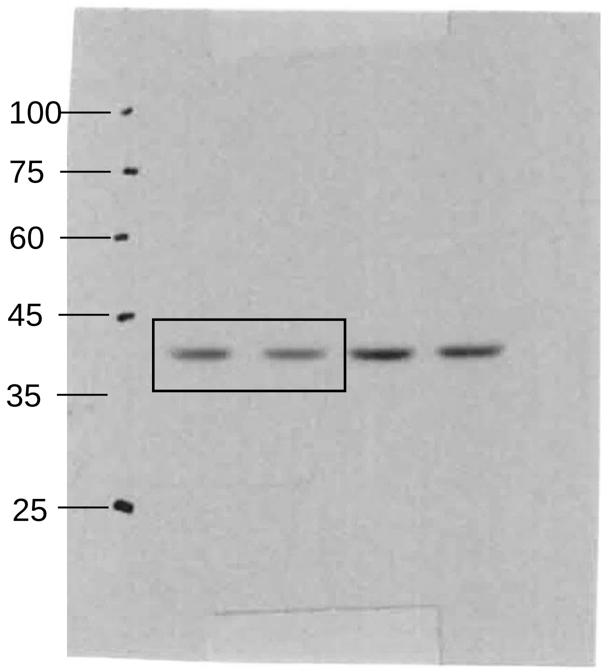

Fig. S5A

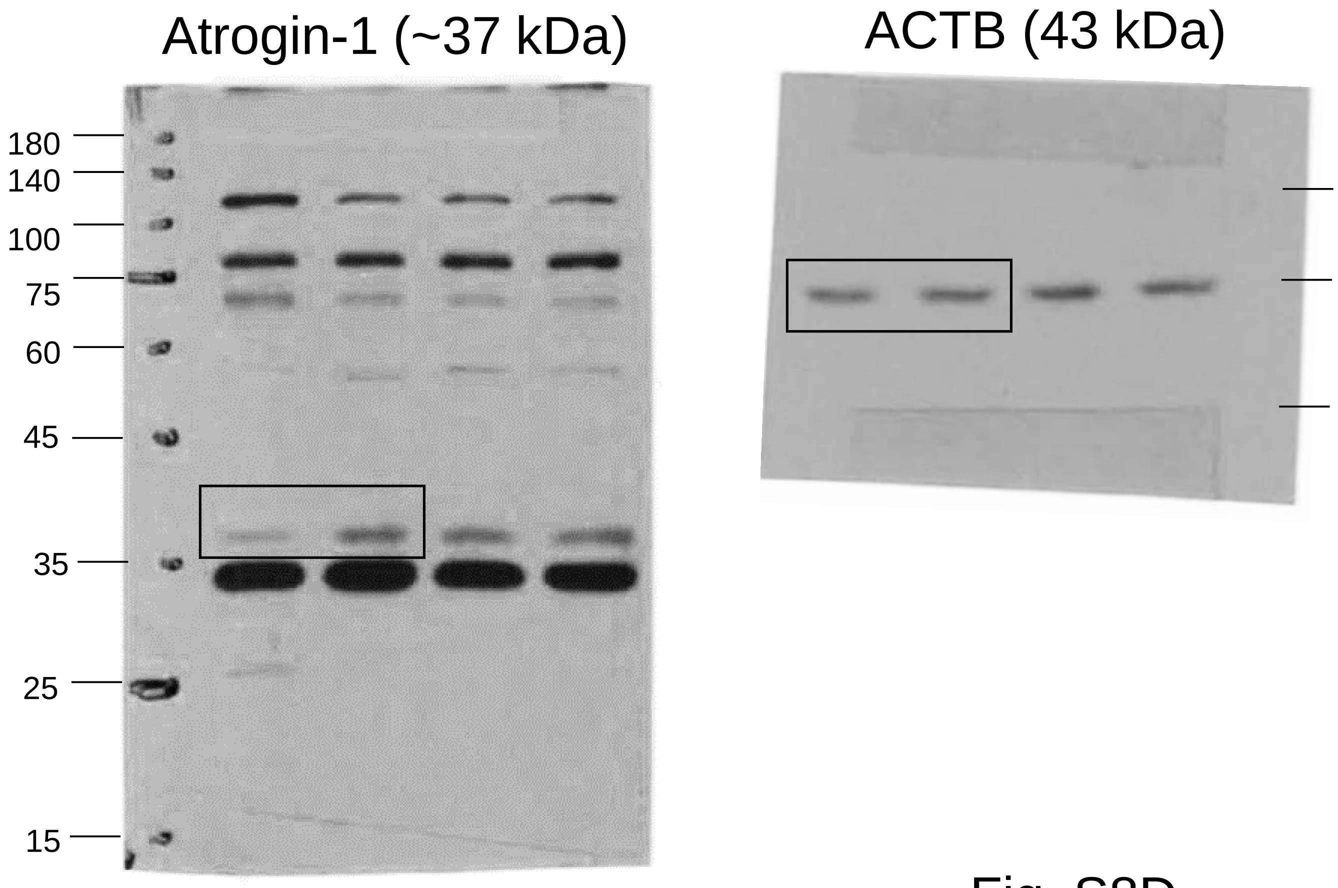

Fig. S5B

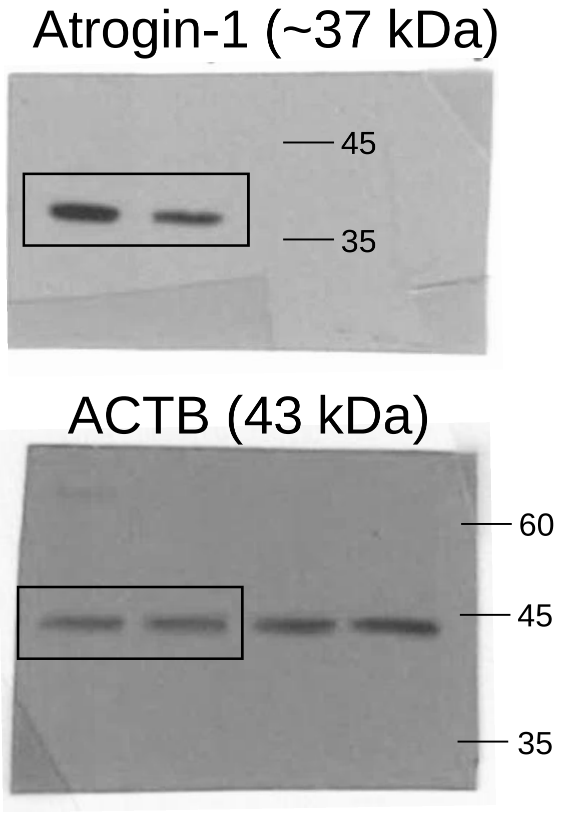

Fig. S8D

Fig. S5B

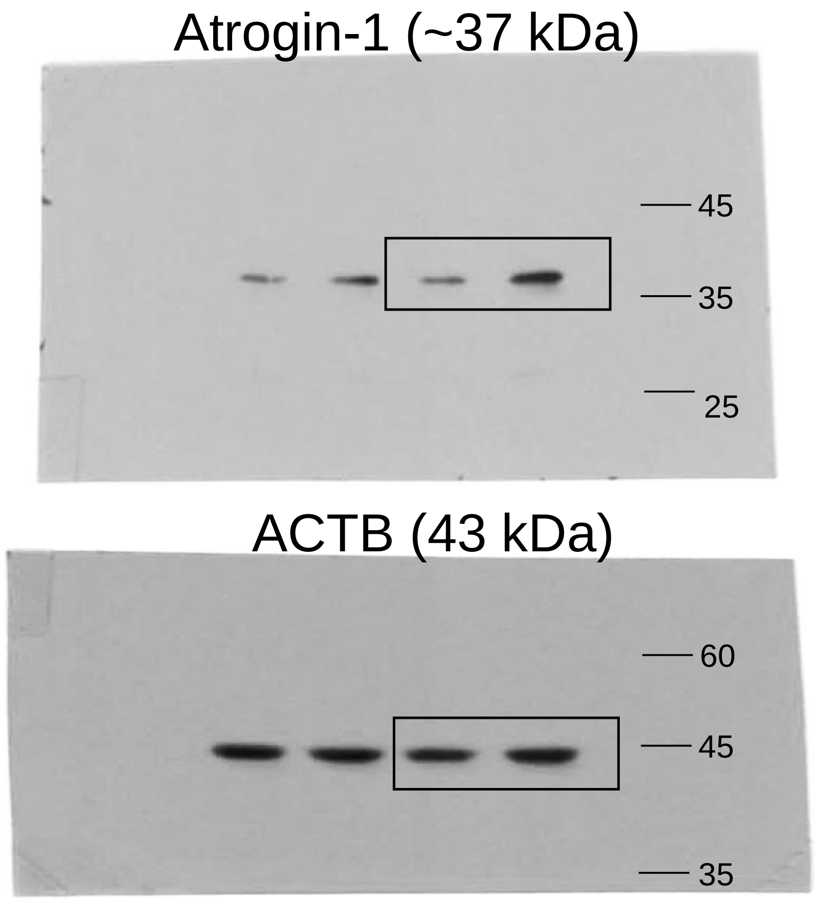

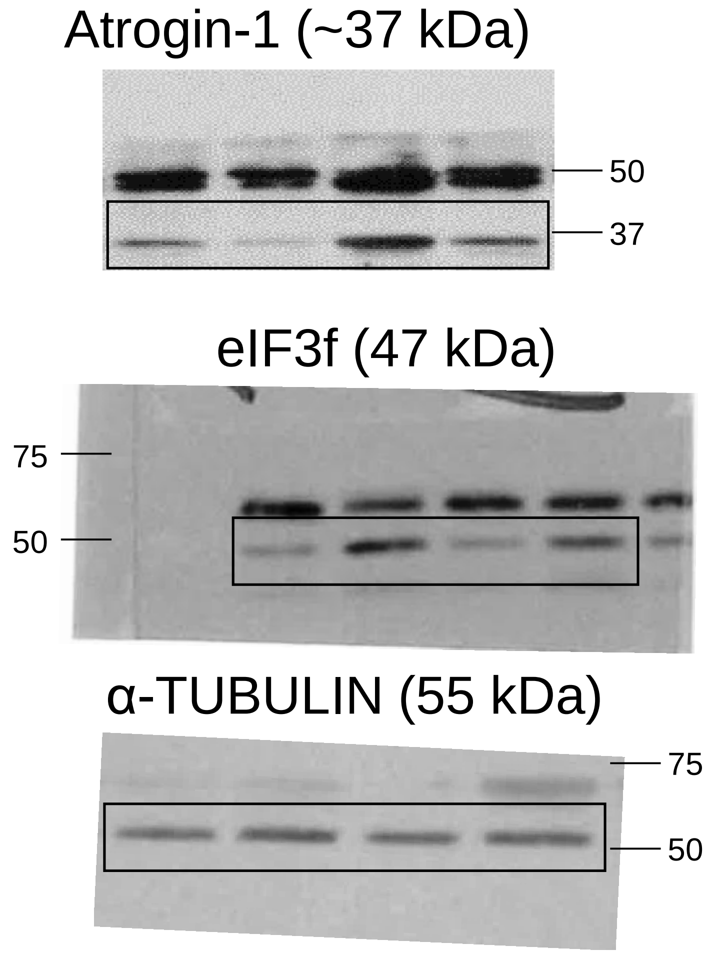

Fig. S9E

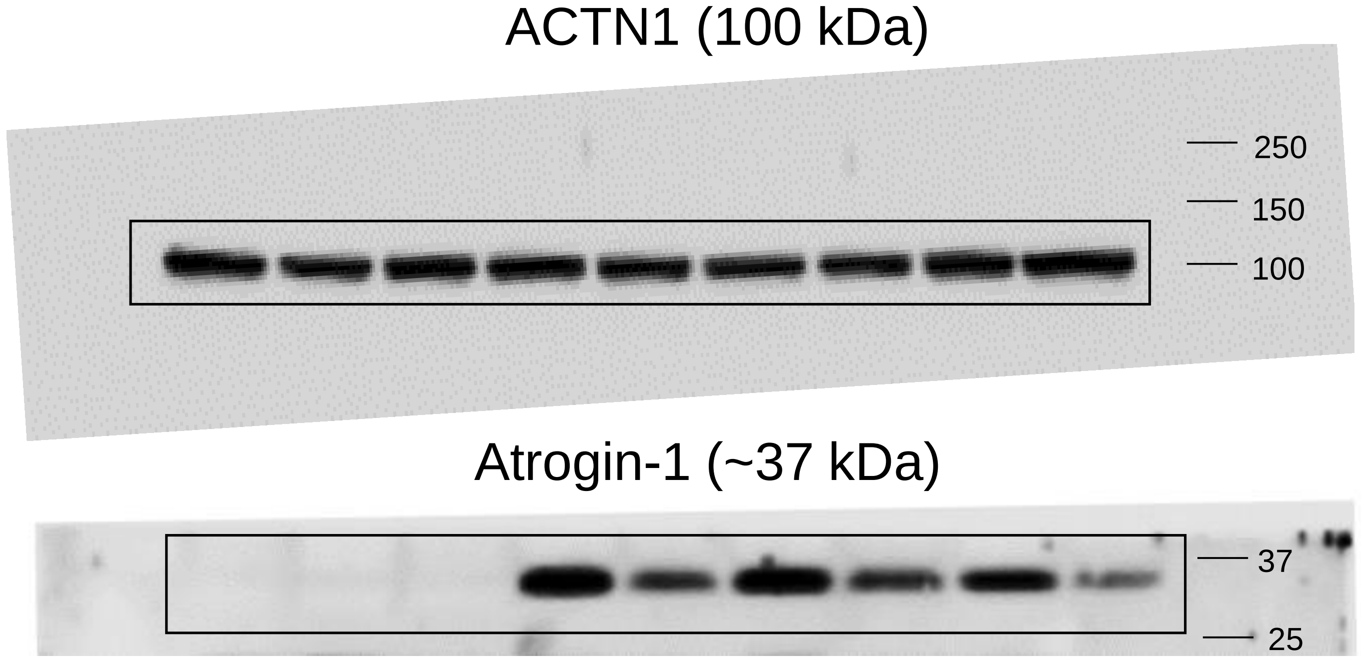

Supplement: Supplementary file 1 — Figure S1. Relative luciferase activity of non‐conserved miRNAs. Relative activity of luciferase reporters bearing the Atrogin‐1 3′ UTR in 293 T cells transfected with the indicated miRNAs. The data are presented as the mean ± SD. *P < 0.05, **P < 0.01, ***P < 0.001. Figure S2. Immunoblot analysis of anabolic and catabolic components in differentiated HSMMs (from a 19‐year‐old donor). Immunoblots of the indicated proteins from differentiated HSMMs (from a 19‐year‐old donor) transfected with the indicated miRNAs. The Atrogin‐1 and eIF3f protein levels were quantified using ImageJ and normalized to α‐tubulin. Figure S3. Expression levels of the top 5 miRNAs inducing the antiatrophic phenotype on C2C12 myotubes in young and aged TA muscle. Relative expression of miR‐668, 376c, 494, 541, and 1197 in young and aged TA muscle (n = 5). The data were normalized to the U6 snRNA level and presented as the mean ± SD (*P < 0.05). Figure S4. Pull‐down of Luciferase 2 containing wild‐type (WT) or deletion mutant (Mut) miR‐376c‐3p binding site in Atrogin‐1 3′ UTR. C2C12 cells were transfected with the indicated luciferase reporters containing wild‐type (WT) or deletion mutant (Mut) miR‐376c‐3p binding sites in the Atrogin‐1 3′ UTR. At 48 h after transfection, Luciferase 2 mRNA was pulled down using ASO (in the presence or absence of biotin) with streptavidin beads, and RT‐qPCR analysis was performed to detect Luciferase 2 mRNA enrichment. The data are presented as the mean ± SD of 3 independent experiments. Figure S5. miR‐376c‐3p inhibits Atrogin‐1 protein content in fully differentiated C2C12 cells and HSMMs. (A, B) Immunoblot analysis of Atrogin‐1 in M‐miR‐376c‐3p‐ and I‐miR‐376c‐3p‐transfected C2C12 cells (A) and HSMMs (B). The results were normalized to ACTB levels. (C) Ratio of protein accumulation normalized by genomic DNA content in differentiated HSMMs transfected with M‐miR‐376c‐3p or control. The data are presented as the mean ± SD. **P < 0.01. Figure S6. Aged mice exhib [file JCSM-11-1336-s001.pdf]
